# An integrated multi-criteria decision framework for outlet mall location selection using fuzzy DEMATEL–DANP–VIKOR

**DOI:** 10.1038/s41598-026-42895-0

**Published:** 2026-03-31

**Authors:** Ming-Han Chiang, Bao-Hui Huang, Cheng-Ru Wu, Jhong-Min Yang, Chin-Fa Tsai, Hung-Wen Lee, Xia-Sui Peng

**Affiliations:** 1 National Tainan Junior College of Nursing, 78, Sec. 2, Minzu Rd., West Central Dist., Tainan City, 700007 Taiwan; 2 Wuyi College, No. 358 Baihua Road, Wuyishan City, 354300 Fujian Province China; 3 Chaoyang University of Technology, No. 168 Jifong E. Rd., Wufong Township, Taichung city, 413310 Taiwan (R.O.C.); 4 National Chiayi University, No.300 Syuefu Rd., Chiayi City, 600355 Taiwan

**Keywords:** Optimal location selection, Diamond model, Fuzzy DANP, VIKOR, Sensitivity analysis, Engineering, Mathematics and computing

## Abstract

Outlet malls, as integrated retail spaces combining shopping, dining, and leisure, have rapidly expanded in global markets, and their location planning involves multiple interconnected, strategic criteria. This study develops an integrated multi-criteria decision-making (MCDM) framework that includes Porter’s diamond model within a fuzzy DEMATEL–DANP–VIKOR approach to support the comparison of outlet location options. Seventeen evaluation indicators were identified through a literature review and a modified Delphi process. The fuzzy DEMATEL method was used to analyze causal relationships among criteria, while the DANP method was used to determine interdependent weights. VIKOR was then applied to rank three candidate locations shortlisted during the initial planning stage in Tainan, followed by sensitivity analysis. The results show that the proposed framework clarifies the patterns of structural influence and preference-sensitive rankings while maintaining stability across different decision preferences. Overall, the framework offers a systematic, theory-based decision-support tool for retail investment and outlet location planning.

## Introduction

With the rapid growth of digital technologies and e-commerce, consumer shopping habits have shifted from prioritizing functionality to valuing experiences, emotional connections, and immersive interactions^1,2^. This change has prompted retailers to reevaluate their store layouts and operational strategies, transforming physical retail spaces from simple transaction points into experiential environments that offer entertainment, social interaction, and emotional engagement. As hybrid retail formats that combine shopping, dining, and leisure, outlet malls have experienced rapid growth worldwide in recent years, becoming important venues for social and recreational activities^3,4^. Their spatial design and tenant mix significantly influence customers’ dwell time, emotional responses, and behavioral reactions^5^.

However, developing outlet malls requires significant capital investment and long-term planning, with location decisions influenced by factors such as land costs, accessibility, market potential, competitive landscape, and policy support. The correct location often determines whether an investment will succeed or fail^6,7^. For multinational companies, outlet mall location strategies must also consider cross-cultural differences, regulatory issues, and regional competition. Without a systematic evaluation framework, decision-making can lead to the misallocation of resources and long-term financial losses^8,9^.

In Taiwan, Mitsui Fudosan has been developing Mitsui Outlet Parks in Linkou, Taichung Port, and Kaohsiung since 2016, demonstrating a strategic approach to cross-border retail investment. As one of Japan’s largest real estate developers, Mitsui’s international expansion exemplifies foreign direct investment (FDI), which entails substantial capital costs, long-term operational risks, and the need to adapt to local market structures, regulations, and cultural preferences^8,10^. Its location strategy targets attracting foot traffic, increasing the number of restaurants, and optimizing circulation design to boost customer dwell time and encourage repeat visits^11^. As of 2022, there are seven outlets in Taiwan, primarily located in the northern region, with fewer in the south. Among them, the outlets in Kaohsiung are far from the city center, and their transportation and regional connectivity need improvement^12^. Based on this situation, Mitsui Head Office decided to open an OUTLET in Tainan to strengthen its presence in the southern market and improve its competitiveness with department stores.

When evaluating Tainan as a potential location, Mitsui must consider various uncertainties, including market structure, transportation infrastructure, and competitive pressure. Traditional retail location theories—such as the Central Place Theory (Christaller, 1933)^13^, the Law of Retail Gravitation (Reilly, 1931)^14^, the Principle of Minimum Differentiation (Hotelling, 1990)^15^, and Rent Theory (Brown, 1993; Krey et al., 2022)^16,17^—explain spatial behavior based on distance, customer flow, and costs. However, these models often assume independence among factors and static market conditions, which makes them inadequate for capturing complex interactions and changing interdependencies in modern outlet location systems^18,19^. Therefore, creating a comprehensive evaluation framework that incorporates spatial, economic, industrial, and policy factors has become a key focus in retail investment research.

In recent years, multi-criteria decision-making (MCDM) methods have been widely used in retail and commercial facility location studies, as they allow for the integration of quantitative indicators (e.g., traffic flow, population density) with qualitative factors (e.g., brand image, experiential atmosphere), thus improving both scientific accuracy and comparability in decision-making^20,21^. In high-investment, high-risk situations, location assessment serves not only as a spatial planning tool but also as a strategic decision-making instrument. The methodological rigor of these analyses greatly influences the efficiency of resource allocation and long-term operational success.

To address these challenges, this study uses Porter’s Diamond Model^22^ as the theoretical foundation to analyze how firms develop competitive advantages through location strategy. The model highlights six factors that influence national and industry competitiveness: factor conditions, demand conditions, firm strategy, structure and rivalry, related and supporting industries, government, and chance. The first four factors are internal elements that firms can actively manage and improve. Meanwhile, the government is an external but influential force that can shape the investment climate through policy measures. In contrast, chance refers to unpredictable external events—such as technological breakthroughs, policy shifts, or global crises—that are beyond managerial control^10,23^.

Since this study emphasizes operational and measurable strategic factors, the uncertainty aspect (opportunity) was excluded from the analytical framework. Instead, the research concentrates on the five controllable dimensions of Porter’s model, creating an integrated evaluation system that employs fuzzy MCDM methods to analyze cause-and-effect relationships and assess the significance of the factors. This hybrid approach enhances both quantitative accuracy and structural clarity, enabling comprehensive ranking and sensitivity analysis of potential locations, and providing a theoretically sound yet practical decision-support framework for businesses and policymakers^8,24^.

Although Porter’s Diamond Model provides a solid theoretical foundation for understanding competitive advantage, it lacks a method for measuring dependencies among its dimensions. To address this, the current study integrates fuzzy DEMATEL, DANP, and VIKOR into a single hybrid MCDM framework. First, fuzzy DEMATEL identifies causal relationships among dimensions and criteria, addresses semantic ambiguity and uncertainty in expert judgments, and produces the Influence Network Relation Map (INRM)^25–27^. Second, DANP translates these causal links into interdependent weights, overcoming the independence assumption of AHP/ANP and capturing the strength and direction of influences among factors^7,28^. Third, VIKOR provides a compromise ranking that balances overall group utility and individual regret, offering a practical solution for decision environments with conflicting and preference-sensitive objectives^29,30^. Finally, a sensitivity analysis assesses the stability and robustness of the results across different preference scenarios, increasing confidence in real-world decision-making.

In summary, this study introduces an integrated "Diamond Model—fuzzy DEMATEL–DANP–VIKOR" framework that combines theoretical principles with quantitative precision. The model methodically identifies the key factors and relationships that impact outlet location competitiveness, offering a solid analytical foundation for evidence-based strategic planning, investment decisions, and policy development in the retail industry.

The contributions of this study are outlined in three main ways. (1) It introduces a multidimensional evaluation framework that combines the Porter Diamond Model with the Fuzzy MCDM method, connecting strategic competitiveness theory with spatial decision analysis. (2) It provides both quantitative and qualitative methods for selecting the optimal location for outlet malls, capable of assessing tangible factors (such as land size and transportation) and intangible factors (like brand awareness and compatibility with the business district). (3) The framework is validated through an empirical case in Taiwan, offering a replicable and transparent tool for international retail investment and spatial planning with policy implications.

The research structure is outlined as follows: Section "Introduction" describes the research background and objectives; Section "Literature review" reviews the literature, including retail location selection theory, consumer behavior in the new retail environment, and Porter’s diamond model along with retail competitive advantage analysis; Section "Methodology" develops the fuzzy DEMATEL–DANP–VIKOR model and establishes evaluation indicators; Section "Case implementation" presents an empirical study using the Tainan outlet mall as an example; and Section "Conclusions and limitations" summarizes the findings, offers management implications, and suggests future research directions.

## Literature review

### Theoretical foundations and evolution of retail location selection

Choosing retail locations is a crucial part of both business strategy and urban planning, as it significantly impacts market reach, customer traffic, and long-term competitiveness^7,9,12^. As retail spaces have shifted from traditional commercial districts to multifunctional areas, such as shopping centers and outlet malls, location theory has evolved from static geographic models to more advanced frameworks that incorporate behavioral economics and spatial analytics^16,31^. Early classical theories, such as Christaller’s Central Place Theory (1933), emphasized the hierarchical distribution of retail hinterlands and service coverage^13^. Reilly’s Law of Retail Gravitation (1931) stated that consumer movement is inversely related to distance and directly related to attraction^14^. Hotelling’s Principle of Minimum Differentiation (1990) suggested that retailers tend to cluster to minimize customer loss^15^. Additionally, rent-based theories suggest that land value reflects consumer potential and spatial hierarchy, with high rents typically indicating high purchasing power and brand concentration^17^.

As urban areas grow more complex and geographic data become more accessible, research on retail location has shifted from static, theoretical models to dynamic, data-driven analyses. Formánek and Sokol (2022) found that geographic visibility and socio-demographic traits have a significant impact on retail success^31^. Erdin and Akbaş (2019) combined Geographic Information Systems (GIS) with the fuzzy TOPSIS method to analyze how spatial interaction and socio-economic factors affect the location of shopping malls^32^. Similarly, Arslan and Ergener (2023) employed space syntax analysis to assess the accessibility and movement efficiency of various mall layout designs^33^. Overall, these studies underscore the crucial role of spatial analytics and geospatial data in contemporary retail location research.

In recent years, Multi-Criteria Decision-Making (MCDM) has become the primary analytical approach in retail location studies. Researchers have combined techniques such as the Analytic Hierarchy Process (AHP), Analytic Network Process (ANP), Technique for Order Preference by Similarity to Ideal Solution (TOPSIS), and VIKOR to improve systematic evaluation and cross-dimensional integration^4,34^. For example, Singh et al. (2020) developed a fuzzy TOPSIS-GRA hybrid model to prioritize convenience store location selection^21^; Alwedyan (2024) and Berumen Calderón et al. (2021) applied the MCDM framework to choose locations for restaurants and themed restaurants, considering factors like cost, service, and market potential^20,35^. Lin et al. (2021) examined location factors for Asian beverage stores by combining fuzzy theory and DEMATEL, demonstrating that the hybrid MCDM framework effectively captures the influence of market conditions and consumer preferences^36^.

From a methodological perspective, integrating fuzzy logic and DEMATEL has proven particularly effective in managing complex interdependencies among criteria. Abikova (2020) and Altuntas and Yilmaz (2016) used fuzzy DEMATEL–ANP models to map causal relationships among decision factors^25,26^. Building on this, Wang and Tzeng (2012) and Büyüközkan et al. (2024) introduced a hybrid framework combining fuzzy DEMATEL, DANP, and VIKOR to improve ranking stability and theoretical consistency^28,29^. Similarly, Tadić et al. (2014) successfully applied a fuzzy DEMATEL–ANP–VIKOR model for selecting urban logistics concepts, confirming its robustness and interpretability^37^.

With advances in machine learning and spatial big data analytics, retail location research has entered a new, data-driven phase. Wang et al. (2024) combined a Variational Graph Autoencoder and Random Forest (VGAE–RF) model for urban shelter site prediction^38^; Liu et al. (2024) used Random Forest to analyze retail distribution patterns^39^; and Ou Yang et al. (2020) employed convolutional neural networks to model spatial competition among retailers^40^. These trends demonstrate how intelligent systems and data science are now essential to evidence-based retail location decision-making.

In summary, retail location theory has shifted from static models based on distance and cost to dynamic frameworks that incorporate consumer behavior, spatial interactions, and MCDM. While this progress is significant, research specifically on outlet-type retail locations remains limited. To address this gap, the current study combines Porter’s Diamond Model of industrial competitiveness with a fuzzy DEMATEL–DANP–VIKOR hybrid MCDM approach to develop a comprehensive location evaluation model for outlet malls. This integrated method combines the theoretical strength of Porter’s model with practical MCDM techniques, providing a reliable framework that enhances understanding of the key competitive factors and their interactions in outlet mall location decisions.

### Consumer behavior in the new retail environment

With rapid advances in digital technology and e-commerce, the global retail industry is undergoing a fundamental shift from a "function-oriented" to an "experience-oriented" approach. Consumers are no longer solely focused on price and product features; instead, they increasingly value emotional connections, social engagement, and immersive shopping experiences^1^. Helm, Kim, and Van Riper (2020) highlight that physical retail spaces are evolving from simple transaction points into versatile experience platforms that combine entertainment, social interaction, and emotional bonds^2^. Elmashhara and Soares (2019) also confirm that mall atmospheres and entertainment amenities significantly boost customers’ emotional responses, extend their visits, and increase their purchase intentions^5^.

In line with this cultural shift, integrated retail spaces that combine shopping, dining, and leisure—such as outlet malls—have become key destinations for experience-based consumption. Wu, Kuang, and Lo (2019) observe that spatial comfort and transportation accessibility strongly influence customer satisfaction and purchase intentions^74^. Bawa, Sinha, and Kant (2019) found that young consumers prefer shopping environments that offer entertainment and social interaction^3^. Singh et al. (2020) emphasize that a destination’s entertainment value and social atmosphere are crucial for attracting customers^21^^.^

With the rise of the “New Retail” concept, integrating online and offline channels has become a crucial strategic move. Physical stores are no longer just points for transactions but serve as primary touchpoints for brand experiences and customer engagement^42,43^. Sung (2022) notes that the accessibility and location of physical spaces significantly affect retail performance^44^, while Sharma et al. (2024) argue that experiential engagement fosters emotional bonds and encourages repeat purchases^45^. Zhou et al. (2024) further observe that impulse buying, and leisure motivation are vital for the success of shopping malls, with space design and atmosphere creation becoming essential strategies^79^. Yiu et al. (2024) redefine the retail catchment area analysis model, emphasizing that geographic distribution analysis based on purchasing power more accurately reflects the competitive advantage of outlet mall locations^47^.

Overall, current consumer shopping behavior is increasingly shaped by trends of "experiential, social, and emotional" consumption. The success of outlet malls depends not only on brand variety and pricing strategies but also on their capacity to provide immersive emotional experiences and foster customer loyalty. When evaluating locations, it is essential to consider both tangible factors—such as transportation access, land conditions, and consumer capacity—and intangible factors—including atmospheric design, experiential quality, and customer psychological responses. This experience-driven consumer behavior approach supports the development of the outlet location evaluation system in this study, which combines Porter’s Diamond Model with fuzzy MCDM methods.

### Porter’s diamond model and retail competitive advantage

Amid the rise of experience-driven consumption and increasing retail competition, traditional location theories—focused mainly on spatial attributes such as distance, rent, and accessibility—have become less sufficient for explaining the complex mechanisms underlying outlet mall location competitiveness. These theories often ignore systemic influences, including industrial interdependence, brand clustering effects, and policy environments that collectively shape retail performance^7,16,48^. To overcome these limitations, this study uses Porter’s Diamond Model^22,23^ as a theoretical framework to examine outlet location competitiveness from a dynamic, systemic perspective.

Porter’s Diamond Model views regional competitiveness as the result of interactions among four key internal factors—factor conditions, demand conditions, firm strategy, structure and rivalry, and related and supporting industries—augmented by government and chance as external influences. Instead of functioning independently, these factors interact through feedback loops that together shape an evolving competitive ecosystem^10,49–51^. The Diamond Model has been widely used in studies of retail, services, and regional competitiveness to provide a structured framework for understanding multi-level and cross-dimensional influences.

1. Factor conditions refer to the essential resources supporting retail investment and operations, such as land availability, labor supply, capital inputs, and transportation infrastructure. Prior studies indicate that favorable land costs and accessibility can boost investment appeal and operational feasibility, while sufficient spatial capacity enables long-term development flexibility^75,19,74^. These factors collectively define the basic production environment in which outlet malls operate.
2. Demand conditions describe the structure and quality of market demand that retail developments face. Population size, purchasing power, income levels, and demographic makeup influence the size and stability of the customer base, while consumption frequency and lifestyle-related demand impact repeat visits and spending habits^76,77^. In the context of new retail, digital engagement and online word-of-mouth also shape demand perceptions and location performance^79^.
3. Firm strategy, structure, and rivalry focus on the strategic positioning of companies and the competitive dynamics within a specific region. Brand awareness, consistent image, and alignment with neighboring business districts help create differentiation and build consumer trust, while competitive intensity and market growth potential impact long-term sustainability^29,54^. Clustering among multiple brands can also produce synergistic effects that boost destination appeal.
4. Related and supporting industries emphasize the importance of industrial linkages and complementary services in enhancing retail competitiveness. Past research on outlet malls and large-scale retail clusters has examined a wide range of supporting factors, including premium retail variety, dining and entertainment options, and logistics-related conditions, which may serve as relevant contextual factors influencing consumer experience, operational efficiency, and agglomeration effects^63,78^. However, the specific significance and measurability of these elements largely depend on the context and differ across decision-making environments.
5. Government functions as an external institutional force shaping the investment environment through infrastructure provision, regulatory frameworks, and policy incentives. Transportation development and urban planning can improve regional accessibility and spatial integration, while fiscal incentives and regulatory support may lower entry barriers and boost investor confidence^44,56,57^. Recent studies have also emphasized sustainability-focused governance as an emerging factor in retail location strategies, though its practical relevance depends on local policy contexts and data availability^58^.
6. Chance refers to external and unpredictable events—such as technological disruptions, policy changes, or global crises—that may affect retail location outcomes beyond the control of firms or governments^59^. Due to their random nature and limited comparability in expert-based multi-criteria frameworks, chance is acknowledged but excluded from the operational model. This study, therefore, focuses on structural, decision-relevant factors during the planning stage to improve the robustness of outlet mall location decisions.

Importantly, Porter’s Diamond Model is not used as a hypothesis-testing framework in this study. Instead, it functions as a theory-driven structural ontology that outlines the main dimensions of outlet location competitiveness and directs the hierarchical arrangement of evaluation criteria. Based on this theoretical framework, subsequent analyses combine a modified Delphi method with fuzzy DEMATEL–DANP–VIKOR techniques to refine context-specific indicators, explore causal relationships among dimensions, and evaluate their relative importance in outlet mall location decisions.

## Methodology

### Construction of outlet location evaluation criteria

To ensure consistency between the theoretical framework and practical implementation, this study used a modified Delphi method to refine the evaluation criteria for outlet mall location selection. Based on the conceptual structure outlined by Porter’s Diamond Model (Section "Porter’s diamond model and retail competitive advantage"), the Delphi process was employed to convert broad theoretical dimensions into context-appropriate and actionable criteria suitable for quantitative multi-criteria decision-making analysis.

Drawing on the literature, an initial set of 32 candidate criteria was identified to represent the potential scope of outlet mall location competitiveness. These candidates were then evaluated by a panel of experts using a seven-point Likert scale for their importance and contextual relevance. Through three rounds of anonymous consultation with structured statistical feedback involving experts from academia, industry, and the public sector, expert opinions gradually converged. Criteria lacking sufficient consensus or operational relevance were systematically revised or excluded according to predefined screening rules, resulting in a final set of 17 criteria. The retained criteria, their dimensional classification, and corresponding literature sources are summarized in Table 1, which forms the basis for the subsequent multi-criteria decision-making analysis. The retained criteria were organized into five primary dimensions corresponding to Porter’s Diamond Model, as follows.

- ***F***: Factor Conditions—Includes capital investment^9,11,28^, labor conditions^31,41,54^ , land area^18,32,46^ , and site visibility^5,60,61^, reflecting the core capacity for development and operation.
- ***D***: Demand Conditions—Includes purchasing power^11,47,53^ , population size^19,31,46^ , and customer demographics^3,45,53,62^ , which collectively influence market potential and the consumer base.
- ***S***: Firm Strategy, Structure, and Rivalry—Includes brand visibility^28,34^, market growth potential^11,29,43^, rivalry^41,48^, and business district compatibility^5,41^, emphasizing strategic positioning and long-term competitiveness.
- ***R***: Related and Supporting Industries—Focuses on boutique suppliers^5,42^, entertainment suppliers^5,62^, and food court suppliers^42,46^, as well as logistics and accommodation infrastructure^10,22,23,55^, which improve customer experience and promote operational synergetic effect.
- ***G***: Government Role—Includes regional development^8,10,56^ and transportation construction^44,46,61^, as well as regulations and incentives like tax breaks and investment subsidies^8,57,63^, and enhances institutional guarantees and investment incentives.

**Table 1:** Dimensions and criteria of the evaluation systems.

Dimensions	Criteria	Explanations	Reference
Factors of production (F)	Capital investment (F1)	Total investment needed for outlet mall construction, including land purchase and development expenses	^9,11,28^
	Labor conditions (F2)	Availability and sufficiency of the workforce, including sales, catering, customer service, and maintenance staff	^31,41,54^
	Land area (F3)	Size of the site, which influences construction costs, spatial layout, and future expansion potential	^18,32,46^
	Site visibility (F4)	Extent of the site’s visibility and accessibility to potential customers	^5,60,61^
Demand conditions (D)	Purchasing power (D1)	Consumers’ purchasing power and willingness to spend money on shopping activities	^11,47,53^
	Population size (D2)	Number of residents in the outlet mall’s primary and secondary trade areas	^19,31,46^
	Customer demographics (D3)	Socioeconomic and behavioral factors that affect brand positioning and customer loyalty	^3,45,53,62^
Strategy and rivalry (S)	Brand visibility (S1)	The level to which the brand is recognized and linked with product quality and design	^28,34^
	Market growth potential (S2)	Expected customer growth rate over a specified period in the target market	^11,29,43^
	Rivalry (S3)	Level of competition in local retail and outlet mall markets	^41,48^
	Business district compatibility (S4)	The alignment level between the outlet mall and nearby business structures affects their attractiveness and dwell time	^5,41^
Related industries (R)	Boutique suppliers (R1)	Vendors providing branded or luxury products to the outlet mall	^5,42^
	Entertainment suppliers (R2)	Providers of leisure and entertainment services, such as cinemas or amusement facilities	^5,62^
	Food court suppliers (R3)	Vendors offer a diverse range of dining and catering options within the outlet mall	^42,46^
Government role (G)	Regional development (G1)	Urban development planning by local authorities influences the potential for outlet expansion and spatial compatibility	^8,10,56^
	Traffic conditions (G2)	Quality of transportation access, including road networks, parking options, and public transit connectivity	^44,46,61^
	Regulations and incentives (G3)	Such as tax cuts, utility discounts, and construction approvals that promote outlet mall investment and sustainable operation	^8,57,63^

Together, these five dimensions include 17 sub-criteria, forming the input framework for MCDM models and establishing a basis for assessing the competitive advantage of outlet mall locations.

### Fuzzy DEMATEL-DANP method

Fuzzy DEMATEL integrates fuzzy set theory with the DEMATEL framework to address semantic ambiguity and uncertainty in expert judgments, making it suitable for complex decision-making problems that lack objective or large-sample quantitative data^59,64^. Recently, fuzzy DEMATEL has been widely employed across various fields, including sustainable project management^8^, freight village site selection^7,25^, refugee camp planning^26^, logistics center site selection^65^, public opinion event assessment^66^, decision-making tool development^67^, renewable energy selection^29^, and recreational comfort evaluation in national parks^68^.

To identify causal relationships and derive dependency-based weights among dimensions and criteria, this study adopts an integrated fuzzy DEMATEL–DANP approach. Fuzzy DEMATEL is used to reveal interaction structures and feedback effects, while DANP extends these causal relationships to calculate global weights within an analytic network framework^28,69^. Detailed mathematical formulations are provided in Appendix A.

#### Fuzzy DEMATEL analysis

*Step 1: Examination of consistency*. A consistency check was performed before the fuzzy transformation using the inconsistency rate specified in Equation (A1), which measures agreement among expert judgments rather than statistical consistency. When the inconsistency rate was below the set threshold of 5%, the expert judgments were deemed acceptable for further fuzzy DEMATEL analysis^68^.

*Step 2: Construction of the fuzzy direct-relation matrix*. Eighteen domain experts assessed the influence levels among dimensions and criteria using a five-point scale from 0 (no influence) to 4 (powerful influence). To minimize semantic ambiguity and subjective variation, linguistic assessments were converted into triangular fuzzy numbers (TFNs) and then defuzzified using the CFCS method proposed by Opricovic and Tzeng (2007)^56^, following the procedures outlined in Appendix A. The fuzzy scales and their associated TFNs are shown in (Table 2). The defuzzified values were then averaged to create the initial fuzzy direct-relation matrix.

**Table 2:** Linguistic variable and triangular fuzzy number.

Linguistic variable	Triangular fuzzy number
No influence (*0*)	(0, 0, 0.25)
Very low influence (*1*)	(0, 0.25, 0.5)
Low influence (*2*)	(0.25, 0.5, 0.75)
High influence (*3*)	(0.5, 0.75, 1.0)
Very high influence (*4*)	(0.75, 1.0, 1.0)

*Step 3: Normalization*. The fuzzy matrix is normalized to the range [0,1] to improve stability and consistency^67,70^.

*Step 4: Establish the total influence matrix and INRM*. Calculate the total influence matrix, $T=(I-N{)}^{-1}N$, where $N$ is the normalized fuzzy direct influence matrix, and ***I*** is the identity matrix. This matrix comprehensively reflects the overall influence level between each surface or indicator, including both direct and indirect effects. Then, construct the INRM based on the influence degrees $H$ and *K* to identify driving and affected factors^25,26,71^. When $H-K\boldsymbol{ }>0$, the surface is classified as part of the “cause group”; if $H-K\boldsymbol{ }<0$, it belongs to the "effect group." This step clarifies the interaction direction and hierarchical structure between surfaces, providing a causal foundation for the subsequent calculation of DANP weights.

#### DANP weight calculation

DANP combines the causal structure of DEMATEL with the network weighting logic of ANP, enabling the simultaneous calculation of inter- and intra-contextual dependency weights^28,69^. Its main principle is to create a hypermatrix from the total influence matrix of DEMATEL and to derive the limit matrix through normalization and matrix iteration to determine the global weights. This approach can reveal both the causal structure and the relative importance of dimensions and sub-criteria simultaneously. Since the weight of each sub-criterion in ANP depends on the weight of its respective dimension, this study further improves the traditional DANP weight transmission method by multiplying the dimension-level weighting matrix by the criterion-level unweighted hypermatrix, enhancing the model’s logical consistency and theoretical soundness. The modified DANP weight calculation involves three steps:

*Step 5: Calculate the dimensions, weight matrix, and limit matrix.*

*Step 6: Calculate the weight matrix for the sub-criterion.*

*Step 7: Perform matrix iteration to determine the limit matrix of the sub-criterion and the global weights.*

#### VIKOR

Among multi-criteria decision-making (MCDM) approaches, TOPSIS selects the preferred alternative based on the shortest distance to an ideal solution^72^. In contrast, VIKOR aims to find a compromise solution by balancing overall group utility and individual regret across conflicting criteria^56^. By combining collective performance and worst-case deviation, VIKOR better captures decision-makers’ trade-offs and risk preferences in complex evaluation scenarios. As a result, VIKOR has been widely integrated with DEMATEL- or ANP-based weighting schemes to develop comprehensive decision frameworks that include causal analysis and ranking. It has been extensively applied in facility location selection^34,58^, sustainable energy assessment^29^, supply chain management^73^, and strategic decision-making problems^74^.

In this study, VIKOR is employed to rank alternative outlet mall locations based on the overall criterion weights derived from the DANP analysis. Following Opricovic and Tzeng (2007)^56^, the process is described as follows.

*Step 1: Construction of the performance (benefit) matrix*

The performance scores of each alternative across all evaluation criteria are compiled to form the decision matrix $X =\{{x}_{ij}\}$, where $i$ represents the alternative, and $j$ represents the evaluation criterion. The global weight of each criterion, ${\omega }_{j}$, is derived from the DANP results.

Aligned with the expert-based evaluation design of this study, all criteria are considered benefit-type indicators, meaning higher values represent better performance. This assumption aligns with the expert rating logic used in the survey, as scores reflect perceived relative advantages rather than objective costs or resource use, and it helps avoid ambiguity during subsequent normalization.

*Step 2: Determination of the ideal and anti-ideal solutions*

For each criterion *j*, the ideal solution ${x}_{{\boldsymbol{i}}}^{+}$ and anti − ideal solution ${x}_{{\boldsymbol{i}}}^{-}$ are defined as:${x}_{{\boldsymbol{i}}}^{+}=\underset{{\boldsymbol{i}}}{\mathrm{max}}{x}_{ij}$, and ${x}_{{\boldsymbol{i}}}^{-}=\underset{{\boldsymbol{i}}}{\mathrm{min}}{x}_{ij}$. Given that all criteria are benefit-oriented, no cost-type transformation is required.

*Step 3: Calculation of group utility and individual regret measures*

The group utility measure ${U}_{i}$ and the individual regret measure ${V}_{i},$ for each alternative *i* are computed.

1 $${U}_{i}={\sum }_{j}{\omega }_{j}\frac{{ x}_{{\boldsymbol{j}}}^{+}-{x}_{ij}}{{ x}_{{\boldsymbol{j}}}^{+}-{ x}_{{\boldsymbol{j}}}^{-}},{V}_{i}=\underset{j}{\mathrm{max}}\{{\omega }_{j}\frac{{ x}_{{\boldsymbol{j}}}^{+}-{x}_{ij}}{{ x}_{{\boldsymbol{j}}}^{+}-{ x}_{{\boldsymbol{j}}}^{-}}\}.$$

Here, ${U}_{i}$ represents the overall deviation of alternative *i* from the ideal solution (group utility loss), while ${V}_{i}$ reflects the maximum regret associated with the weakest-performing criterion.

*Step 4: computation of the compromise index*

The overall compromise index ${{\boldsymbol{Q}}}_{{\boldsymbol{i}}}$ is calculated as:

2 $${{\boldsymbol{Q}}}_{{\boldsymbol{i}}}={\boldsymbol{v}}\frac{{U}_{i}-\underset{{\boldsymbol{i}}}{\mathrm{min}}{U}_{i}}{\underset{{\boldsymbol{i}}}{\mathrm{max}}{U}_{i}-\underset{{\boldsymbol{i}}}{\mathrm{min}}{U}_{i}}+(1-{\boldsymbol{v}})\frac{{V}_{i}-\underset{{\boldsymbol{i}}}{\mathrm{min}}{V}_{i}}{\underset{{\boldsymbol{i}}}{\mathrm{max}}{V}_{i}-\underset{{\boldsymbol{i}}}{\mathrm{min}}{V}_{i}},$$

where ${\boldsymbol{v}}$ ∈ [0,1]is the compromise parameter reflecting the relative importance of group utility versus individual regret. Following common practice and prior studies^56^, this study adopts ${\boldsymbol{v}} = 0.5$, assigning equal importance to both components.

*Step 5: Ranking and compromise solution validation*

Alternatives are ranked in ascending order of ${{\boldsymbol{Q}}}_{{\boldsymbol{i}}}$, with smaller values indicating better compromise performance. The top-ranked alternative is considered acceptable if the following conditions are satisfied:

- C1 (Acceptable advantage): $Q(a_{2} ) - Q(a_{1} )~ \ge ~1/(m - 1)$, where *m* is the number of alternatives.
- C2 (Acceptable stability): The best-ranked alternative also ranks first in terms of either ${U}_{i}$ or ${V}_{i}$.

If only one condition is met, the first two alternatives are proposed as compromise solutions; if either condition is satisfied, the alternative with the smallest ${{\boldsymbol{Q}}}_{{\boldsymbol{i}}}$ is selected.

By integrating DANP-derived dependency weights with VIKOR’s compromise-ranking method, this study creates a transparent decision process that involves identifying causal structures, weighting dependencies, and assessing compromises. This combined approach enhances the interpretability, stability, and consistency of outcomes in outlet mall location selection.

### Sensitivity analysis

The final ranking of alternatives mainly depends on the relative importance given to evaluation dimensions and criteria. Since these weights are determined by expert judgment through the DANP process, some level of uncertainty is unavoidable. To evaluate the stability and reliability of the ranking results, this study performs a sensitivity analysis using decision-support software (Expert Choice) to examine how the VIKOR-based ranking responds to systematic variations in the DANP-derived criterion weights^57,61^.

Instead of testing parameters within the VIKOR algorithm, the sensitivity analysis concentrates on weight variation, since uncertainty in expert-based MCDM frameworks mainly arises from the elicitation of criterion weights rather than the deterministic ranking process itself. In line with standard practice^56^, the VIKOR compromise parameter ${\boldsymbol{v}}$ is set to 0.5, assigning equal importance to group utility and individual regret. Three types of sensitivity experiments are performed:

1. Weight perturbation: Single- and multi-factor perturbations of ± 5%, ± 10%, and ± 20% are applied to primary and secondary criteria. Weights are re-normalized to sum to 1, and changes in rankings are observed.
2. Equal-weight baseline: All criteria are assigned equal weights to evaluate the model’s dependence on the original DANP-derived structure^28^.
3. Scenario simulation: Market scenarios such as “demand growth,” “transportation improvement,” and “intensified competition” are simulated by adjusting relevant weights, and the resulting rankings are compared for consistency^28,29^.

For each experiment, the shifts in ranking and the stability of the top-ranked alternatives are assessed. The sensitivity results are also compared with the uncertainty bounds indicated by DANP weights to validate the credibility, interpretability, and structural robustness of the final decision outcomes^28,29^.

## Case implementation

This chapter presents a case study of Mitsui Fudosan’s outlet investment project in Tainan City, using an established evaluation framework with five dimensions and 17 sub-criteria. The fuzzy DEMATEL–DANP model examines the causal relationships and interdependencies among these criteria. Outlet mall development demands substantial capital investment and long-term infrastructure, with location decisions influenced by factors such as land costs, transportation access, consumer potential, the competitive landscape, and policy support^6,7^. For multinational firms, location choices also involve institutional risks and structural differences within local markets. Without a systematic decision-making framework, investment mistakes and operational risks may rise.

### Case background

This study explores Mitsui Fudosan’s planned retail outlet investment in Tainan City. Since 2016, the group has established Mitsui Outlet Parks in Linkou and Taichung Port. In 2022, preliminary site assessments began for a new outlet project in southern Taiwan as part of its regional expansion strategy.

**Fig. 1:**
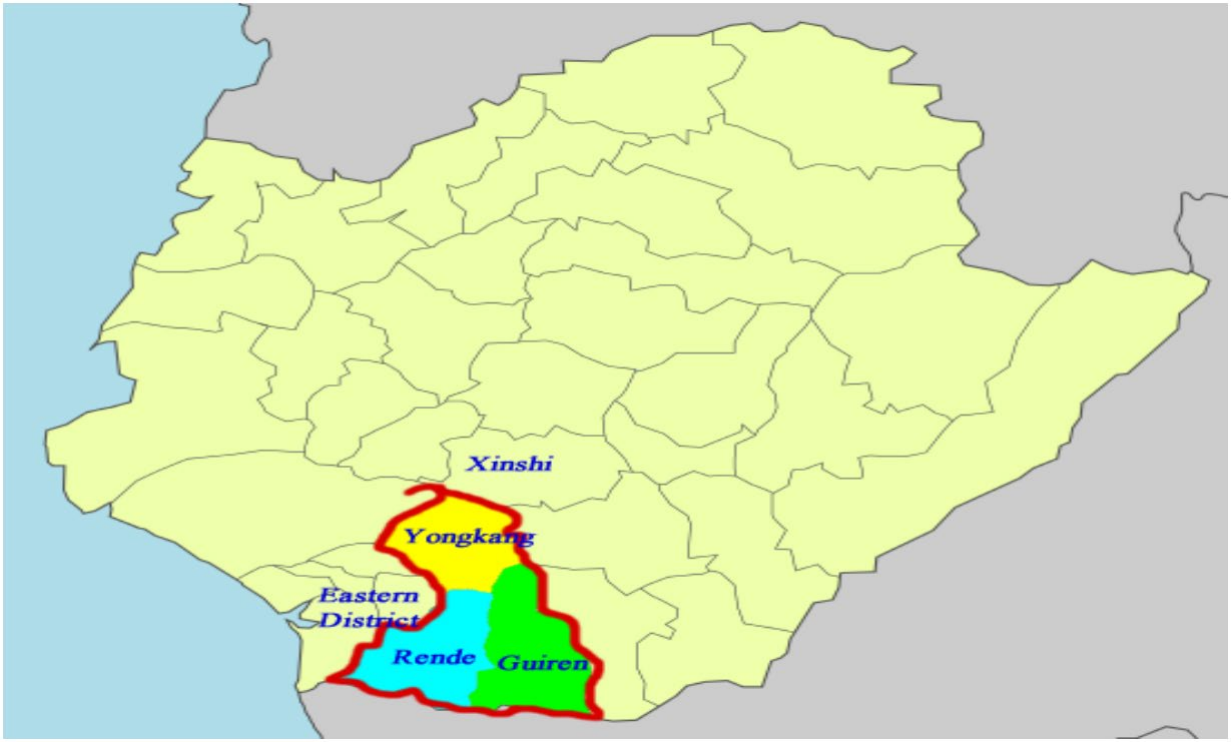
Regional map for the outlet mall, showing locations in the Yongkang District, Guiren District, and Rende District.

During this initial planning phase, three candidate locations—Yongkang District, Rende District, and Guiren District— were publicly identified and discussed as potential sites (Fig. 1). These options were not arbitrarily chosen by the researchers but reflect a realistic, decision-limited shortlist considered during the developer’s preliminary evaluation process. Therefore, the purpose of this study is not to conduct an exhaustive search for all possible outlet locations in Tainan City but to provide a structured comparative assessment of feasible candidates within real-world investment conditions.

Each of the three districts exhibits unique characteristics in terms of population structure, land availability, transportation access, and policy support, making them suitable for multi-criteria decision analysis. Yongkang District has the largest population and a well-developed commercial environment, but faces limitations related to restricted land resources and high development density. Rende District offers moderate land availability and transportation connectivity. At the same time, Guiren District, which hosts the Tainan High-Speed Rail station, provides the largest developable land area, relatively lower competitive pressure, and stronger policy support.

Based on data from the Tainan City Household Registration Office (Table 3), Guiren District has the largest land area, followed by Rende District. In contrast, Yongkang District has the highest population density and consumption potential. However, Yongkang’s dense urban layout and land scarcity could lead to higher infrastructure and development costs. Overall, these three districts showcase contrasting development conditions and strategic trade-offs, making them suitable candidates for applying the fuzzy DEMATEL–DANP–VIKOR framework to aid outlet location decisions.

**Table 3:** Data on land, population size, and density.

	Population	Land area	Population density	
Yongkang District	233,816	40.2753 *km*^2^	**5,805**	
Guiren District	67,916	**55.7913*** km*^2^	1,217	**HSR Tainan Station**
Rende District	76,599	50.7664 *km*^2^	1,509	

### Expert involvement and questionnaire development

An expert panel of 18 members was assembled for this study, comprising specialists from academia, the department store industry, commercial real estate, and the public sector. All members possessed over 15 years of professional experience in their respective fields and had no formal affiliation with Mitsui Fudosan or direct involvement in the specific site selection decision examined. This panel size aligns with the commonly accepted range for MCDM-based studies and adheres to established methodological guidelines for expert judgment research^61^.

Initially, a modified Delphi method was employed to identify the evaluation criteria. This process spanned three rounds, retaining criteria only if they satisfied predefined consensus rules: an interquartile range (IQR) of < 0.5 and a mean importance score of > 5 on a seven-point scale. Ultimately, a final set of 17 criteria was confirmed.

These 17 criteria and their five corresponding dimensions were subsequently incorporated into the fuzzy DEMATEL questionnaire. Because this survey instrument was developed specifically for this study by the authors—derived directly from the literature review and the modified Delphi process—no proprietary third-party questionnaires were utilized, and no external permissions were required for publication. Participation in the survey was strictly voluntary. At the beginning of the questionnaire, a detailed explanation of the research objectives and data usage policies was provided; the completion and submission of the survey by the experts thus constituted their implied informed consent to participate in this study.

Following data collection, the expert judgment scores underwent an agreement-based consistency check (Eq. A1). The survey process adhered to the ethical principles and guidelines monitored by the Office of Research and Development at Chaoyang University of Technology. Responses were accepted for fuzzy conversion and defuzzification only if the inconsistency rate fell below the 5% threshold, ensuring an acceptable level of systemic agreement and reliability among the panel. Furthermore, the same expert panel provided performance evaluations for the three candidate locations—Yongkang, Rende, and Guiren—which served as the input for the VIKOR compromise ranking. Employing this panel-continuity method ensured consistent interpretation across criterion definition, structural influence analysis, and preference-based evaluation, rendering it highly suitable for pre-investment planning scenarios characterized by limited observable data^1,29^.

### Empirical analysis of the INRM based on fuzzy DEMATEL

To identify the causal relationships among factors influencing outlet mall location selection, experts were asked to perform pairwise comparisons of the five dimensions and the seventeen sub-criteria, thereby creating initial direct-relation matrices of sizes 5 × 5 and 17 × 17. Before beginning the fuzzy DEMATEL calculations, a consistency check was performed on the expert judgments using Eq. A1, and the revision process was halted only when the inconsistency rate fell below the preset threshold of 5%. The results show that the inconsistency rates at the dimension and sub-criteria levels were 1.2 and 2.3%, respectively, both well below the threshold, indicating an acceptable level of agreement among experts.

**Fig. 2:**
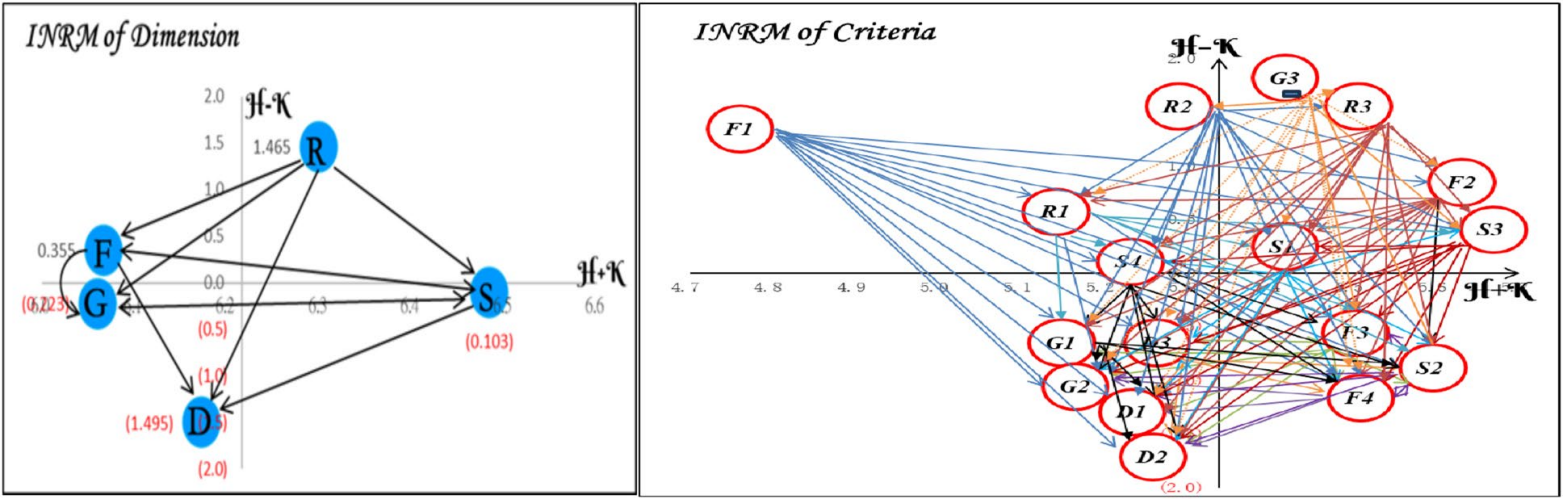
Influential network-relationship map within the system.

After applying Equations (A2)–(A8) in Appendix A, the initial direct-relation matrices underwent fuzzification, defuzzification, normalization, and matrix operations to derive the total influence matrices at both the dimension and sub-criterion levels (Tables 4, 5, 6, 7, 8, 9). To emphasize the most significant influence relationships, the average value of each matrix was used as the threshold. The prominence and causal roles of each factor were then assessed, and an Influence Network Relation Map (INRM) was developed, with the horizontal axis indicating prominence and the vertical axis indicating causal effect (Fig. 2).

**Table 4:** Initial fuzzy influence matrix of dimension.

*Dimension*	***F***	***D***	***S***	***R***	***G***
*F*	0.000	0.708	0.584	0.409	0.608
*D*	0.438	0.000	0.486	0.241	0.417
*S*	0.556	0.703	0.000	0.464	0.554
*R*	0.665	0.840	0.696	0.000	0.659
*G*	0.336	0.567	0.590	0.526	0.000

**Table 5:** Initial fuzzy influence matrix of criteria.

*Criteria*	***F1***	***F2***	***F3***	***F4***	***D1***	***D2***	***D3***	***S1***	***S2***	***S3***	***S4***	***R1***	***R2***	***R3***	***G1***	***G2***	***G3***
*F1*	0.000	0.545	0.633	0.695	0.585	0.858	0.733	0.583	0.765	0.555	0.725	0.585	0.460	0.440	0.760	0.780	0.508
*F2*	0.528	0.000	0.668	0.733	0.860	0.918	0.765	0.610	0.780	0.658	0.640	0.605	0.490	0.455	0.780	0.815	0.530
*F3*	0.390	0.408	0.000	0.640	0.690	0.710	0.608	0.458	0.635	0.538	0.508	0.458	0.315	0.285	0.635	0.658	0.390
*F4*	0.290	0.568	0.530	0.000	0.605	0.633	0.530	0.385	0.590	0.490	0.420	0.393	0.235	0.183	0.558	0.585	0.293
*D1*	0.190	0.383	0.503	0.608	0.000	0.533	0.433	0.468	0.545	0.460	0.445	0.258	0.130	0.245	0.458	0.495	0.260
*D2*	0.133	0.368	0.473	0.560	0.495	0.000	0.420	0.358	0.510	0.383	0.423	0.240	0.093	0.310	0.440	0.465	0.235
*D3*	0.243	0.493	0.633	0.668	0.583	0.615	0.000	0.558	0.668	0.543	0.473	0.335	0.205	0.358	0.533	0.558	0.307
*S1*	0.458	0.535	0.668	0.715	0.758	0.780	0.683	0.000	0.695	0.545	0.508	0.530	0.490	0.543	0.290	0.743	0.433
*S2*	0.308	0.390	0.558	0.618	0.633	0.660	0.560	0.440	0.000	0.460	0.440	0.420	0.408	0.383	0.583	0.605	0.348
*S3*	0.495	0.558	0.755	0.780	0.778	0.803	0.708	0.605	0.765	0.000	0.533	0.553	0.485	0.473	0.743	0.760	0.423
*S4*	0.418	0.465	0.553	0.633	0.708	0.735	0.635	0.533	0.618	0.540	0.000	0.495	0.409	0.383	0.660	0.683	0.380
*R1*	0.435	0.493	0.723	0.755	0.743	0.758	0.660	0.568	0.740	0.543	0.648	0.000	0.468	0.410	0.633	0.658	0.440
*R2*	0.528	0.628	0.780	0.835	0.918	0.958	0.783	0.655	0.808	0.633	0.783	0.635	0.000	0.568	0.790	0.783	0.493
*R3*	0.583	0.560	0.808	0.858	0.958	0.985	0.803	0.683	0.840	0.615	0.840	0.660	0.618	0.000	0.808	0.820	0.508
*G1*	0.243	0.458	0.553	0.618	0.558	0.583	0.483	0.540	0.585	0.510	0.330	0.308	0.418	0.473	0.000	0.530	0.233
*G2*	0.190	0.440	0.530	0.585	0.530	0.560	0.453	0.483	0.553	0.485	0.315	0.285	0.310	0.338	0.490	0.000	0.208
*G3*	0.333	0.790	0.808	0.890	0.660	0.690	0.585	0.833	0.858	0.783	0.808	0.933	0.865	0.808	0.605	0.635	0.000

**Table 6:** Normalized fuzzy influence matrix of dimension.

Dimension	***F***	***D***	***S***	***R***	***G***
*F*	0.000	0.064	0.053	0.037	0.055
*D*	0.040	0.000	0.044	0.022	0.038
*S*	0.050	0.064	0.000	0.042	0.050
*R*	0.060	0.076	0.063	0.000	0.060
*G*	0.030	0.051	0.053	0.048	0.000

**Table 7:** Normalized fuzzy influence matrix of criteria.

*Criteria*	***F1***	***F2***	***F3***	***F4***	***D1***	***D2***	***D3***	***S1***	***S2***	***S3***	***S4***	***R1***	***R2***	***R3***	***G1***	***G2***	***G3***
*F1*	0.000	0.046	0.053	0.058	0.049	0.072	0.061	0.049	0.064	0.046	0.061	0.049	0.039	0.037	0.064	0.065	0.042
*F2*	0.044	0.000	0.056	0.061	0.072	0.077	0.064	0.051	0.065	0.055	0.054	0.051	0.041	0.038	0.065	0.068	0.044
*F3*	0.033	0.034	0.000	0.054	0.058	0.059	0.051	0.038	0.053	0.045	0.042	0.038	0.026	0.024	0.053	0.055	0.033
*F4*	0.024	0.048	0.044	0.000	0.051	0.053	0.044	0.032	0.049	0.041	0.035	0.033	0.020	0.015	0.047	0.049	0.024
*D1*	0.016	0.032	0.042	0.051	0.000	0.045	0.036	0.039	0.046	0.039	0.037	0.022	0.011	0.021	0.038	0.041	0.022
*D2*	0.011	0.031	0.040	0.047	0.041	0.000	0.035	0.030	0.043	0.032	0.035	0.020	0.008	0.026	0.037	0.039	0.020
*D3*	0.020	0.041	0.053	0.056	0.049	0.051	0.000	0.047	0.056	0.045	0.040	0.028	0.017	0.030	0.045	0.047	0.026
*S1*	0.038	0.045	0.056	0.060	0.063	0.065	0.057	0.000	0.058	0.046	0.042	0.044	0.041	0.045	0.024	0.062	0.036
*S2*	0.026	0.033	0.047	0.052	0.053	0.055	0.047	0.037	0.000	0.039	0.037	0.035	0.034	0.032	0.049	0.051	0.029
*S3*	0.041	0.047	0.063	0.065	0.065	0.067	0.059	0.051	0.064	0.000	0.045	0.046	0.041	0.040	0.062	0.064	0.035
*S4*	0.035	0.039	0.046	0.053	0.059	0.062	0.053	0.045	0.052	0.045	0.000	0.041	0.034	0.032	0.055	0.057	0.032
*R1*	0.036	0.041	0.060	0.063	0.062	0.063	0.055	0.048	0.062	0.045	0.054	0.000	0.039	0.034	0.053	0.055	0.037
*R2*	0.044	0.053	0.065	0.070	0.077	0.080	0.066	0.055	0.068	0.053	0.066	0.053	0.000	0.048	0.066	0.066	0.041
*R3*	0.049	0.047	0.068	0.072	0.080	0.082	0.067	0.057	0.070	0.051	0.070	0.055	0.052	0.000	0.068	0.069	0.042
*G1*	0.020	0.038	0.046	0.052	0.047	0.049	0.040	0.045	0.049	0.043	0.028	0.026	0.035	0.040	0.000	0.044	0.019
*G2*	0.016	0.037	0.044	0.049	0.044	0.047	0.038	0.040	0.046	0.041	0.026	0.024	0.026	0.028	0.041	0.000	0.017
*G3*	0.028	0.066	0.068	0.075	0.055	0.058	0.049	0.070	0.072	0.066	0.068	0.078	0.072	0.068	0.051	0.053	0.000

**Table 8:** Total-influence matrix of dimension.

Dimension	***F***	***D***	***S***	***R***	***G***
*F*	0.466	**0.826**	**0.708**	0.521	**0.689**
*D*	0.472	0.457	0.537	0.370	0.502
*S*	**0.627**	**0.822**	0.536	0.532	**0.673**
*R*	**0.755**	**0.990**	**0.849**	0.478	**0.810**
*G*	0.534	**0.738**	**0.663**	0.516	0.468

Threshold α = 0.622. Values greater than the threshold are shown in bold.

**Table 9:** Total-influence matrix of criteria.

*Criteria*	***F1***	***F2***	***F3***	***F4***	***D1***	***D2***	***D3***	***S1***	***S2***	***S3***	***S4***	***R1***	***R2***	***R3***	***G1***	***G2***	***G3***
*F1*	0.084	**0.163**	**0.199**	**0.219**	**0.209**	**0.239**	**0.202**	**0.175**	**0.221**	**0.174**	**0.185**	**0.157**	0.129	0.133	**0.204**	**0.217**	0.129
*F2*	0.131	0.126	**0.210**	**0.231**	**0.239**	**0.253**	**0.212**	**0.184**	**0.230**	**0.189**	**0.185**	**0.164**	0.136	0.139	**0.213**	**0.228**	0.135
*F3*	0.100	0.130	0.121	**0.184**	**0.186**	**0.195**	**0.165**	0.141	**0.180**	0.148	0.143	0.126	0.100	0.102	**0.167**	**0.178**	0.103
*F4*	0.084	0.130	0.149	0.117	0.164	**0.172**	0.145	0.123	**0.161**	0.132	0.124	0.110	0.084	0.085	0.147	**0.157**	0.087
*D1*	0.069	0.106	0.134	0.152	0.102	0.150	0.125	0.118	0.144	0.118	0.115	0.090	0.069	0.081	0.127	0.137	0.077
*D2*	0.060	0.099	0.124	0.140	0.134	0.099	0.117	0.103	0.134	0.106	0.107	0.084	0.061	0.081	0.119	0.128	0.070
*D3*	0.084	0.130	**0.164**	**0.178**	**0.170**	**0.180**	0.110	0.142	**0.175**	0.142	0.134	0.111	0.087	0.103	0.152	**0.163**	0.092
*S1*	0.115	0.153	**0.191**	**0.208**	**0.210**	**0.220**	**0.188**	0.119	**0.203**	**0.163**	**0.159**	0.145	0.124	0.133	**0.157**	**0.202**	0.117
*S2*	0.090	0.124	**0.159**	**0.175**	**0.175**	**0.184**	0.155	0.134	0.123	0.136	0.133	0.118	0.103	0.106	**0.157**	**0.167**	0.096
*S3*	0.124	**0.163**	**0.208**	**0.225**	**0.223**	**0.234**	**0.199**	**0.176**	**0.220**	0.129	**0.169**	0.154	0.130	0.135	**0.201**	**0.215**	0.122
*S4*	0.107	0.141	**0.173**	**0.192**	**0.197**	**0.207**	**0.175**	0.154	**0.188**	0.155	0.110	0.135	0.112	0.115	**0.177**	**0.188**	0.107
*R1*	0.115	0.153	**0.198**	**0.215**	**0.212**	**0.222**	**0.189**	**0.167**	**0.210**	**0.166**	**0.172**	0.104	0.124	0.125	**0.186**	**0.199**	0.119
*R2*	0.137	**0.184**	**0.229**	**0.250**	**0.255**	**0.268**	**0.224**	**0.196**	**0.244**	**0.196**	**0.205**	**0.175**	0.103	**0.155**	**0.224**	**0.236**	0.139
*R3*	0.144	**0.183**	**0.236**	**0.258**	**0.263**	**0.276**	**0.230**	**0.203**	**0.251**	**0.199**	**0.214**	**0.180**	**0.155**	0.112	**0.230**	**0.244**	0.143
*G1*	0.083	0.125	0.154	0.170	**0.165**	**0.173**	0.145	0.138	**0.165**	0.136	0.121	0.106	0.101	0.109	0.106	**0.157**	0.084
*G2*	0.072	0.115	0.142	0.156	0.151	**0.159**	0.133	0.124	0.151	0.125	0.110	0.097	0.086	0.092	0.135	0.104	0.076
*G3*	0.130	**0.205**	**0.242**	**0.266**	**0.248**	**0.261**	**0.220**	**0.219**	**0.259**	**0.216**	**0.217**	**0.207**	**0.179**	**0.180**	**0.220**	**0.237**	0.106

Threshold α = 0.157. Values greater than the threshold are shown in bold.

As shown in Table 10 and Fig. 2, Dimension ***S*** had the highest prominence, emphasizing its strong connection within the system.*** R*** and ***F*** exhibited positive relation values ($H-\mathbf{K}>0$), indicating they are driving factors. In contrast, ***D***, ***G***, and ***S*** displayed negative relation values ($H-\mathbf{K}<0$), functioning as response factors. This suggests that the system is mainly influenced by resource and industry factors, with market and policy factors responding downstream.

**Table 10:** Centrality and causal relationship of dimensions and criteria.

Dimension	$H$	$\mathbf{K}$	$H+\mathbf{K}$	$H-\mathbf{K}$	*Group*	*Criteria*	$H$	$\mathbf{K}$	$H+\mathbf{K}$	$H-\mathbf{K}$	*Group*
Factors of production (***F***)	3.211	2.856	6.066 (4)	0.355	Cause	***F1***	3.038	1.727	4.765 (17)	1.311	Cause
						***F2***	3.203	2.432	5.635 (2)	0.771	Cause
						***F3***	2.468	3.036	5.504 (6)	-0.568	Affected
						***F4***	2.170	3.337	5.506 (5)	-1.167	Affected
Demand conditions (***D***)	2.338	3.833	6.172 (3)	-1.495	Affected	***D1***	1.916	3.302	5.218 (13)	-1.387	Affected
						***D2***	1.769	3.493	5.262 (10)	-1.724	Affected
						***D3***	2.316	2.934	5.25(11)	-0.619	Affected
Firm strategy, structure and rivalry (***S***)	3.191	3.293	6.484 (1)	-0.103	Affected	***S1***	2.807	2.615	5.422 (7)	0.192	Cause
						***S2***	2.337	3.257	5.594 (3)	-0.921	Affected
						***S3***	3.026	2.630	5.655 (1)	0.396	Cause
						***S4***	2.632	2.604	5.236 (12)	0.028	Cause
Related and supporting industries (***R***)	3.882	2.417	6.99(2)	1.465	Cause	***R1***	2.875	2.263	5.137 (16)	0.612	Cause
						***R2***	3.420	1.881	5.301 (9)	1.539	Cause
						***R3***	3.522	1.987	5.509 (4)	1.535	Cause
Government role (***G***)	2.919	3.141	6.06.(5)	-0.223	Affected	***G1***	2.238	2.919	5.156 (15)	-0.681	Affected
						***G2***	2.029	3.158	5.186 (14)	-1.129	Affected
						***G3***	3.612	1.802	5.414 (8)	1.811	Cause

At the sub-criterion level, ***S3*** showed the highest prominence, followed by ***F2***, ***S2***, ***R3***, and F4. These are key nodes with strong interaction effects within the system. Conversely, ***F1***, ***R1***, and ***G1*** had a lower impact. Causality analysis identified ***S3***, ***F2***, ***R3***, ***S1***, ***G3***, and ***R2*** as driving indicators with upstream influence, while ***S2***, ***F4***, ***F3***, ***D1–D3***, and ***G1–G2*** are reactive indicators sensitive to environmental changes. Overall, three structural categories emerge: (1) Driving factors—controllable strategic elements such as capital, labor, brand, competition, and policy; (2) Response factors—exogenous conditions like demographics, traffic, and purchasing power; (3) Central nodes—***S3***, ***F2***, and ***R3***, which exhibit the highest systemic interactivity. These findings align with the system-effect principle proposed by Wang & Tzeng (2012)^28^ and Porter’s Diamond Model (1998a)^22^, confirming that driving dimensions are central to systemic performance.

The INRM further highlights several key causal pathways that define interdimensional interactions. Three main relationships are summarized below:

1. ***F*** → ***D***, ***G***: Factor conditions stimulate demand growth and provoke policy responses. F encourages regional demand expansion by enhancing infrastructure and human conditions^52,53^, thus attracting government investment in transportation and policy initiatives, creating a positive feedback loop of "market-policy"^44,63^.

2. ***S3*** → ***F2-F4***, ***D1-D3***, ***G1-G2***: Competitive pressure influences market and strategic structures. High competition levels lead to changes in brand portfolios and investments, resulting in market restructuring^6^.

3. ***G3*** → Nearly the entire system: Policy incentives serve as cross-level mediators. Fiscal incentives, investment subsidies, and redevelopment programs boost capital flows, infrastructure, and mobility, thereby indirectly influencing firm strategies and market dynamics. This systemic catalytic role of government aligns with previous findings on policy-driven spatial development and institutional leverage in location choices^28,63^.

Overall, the INRM reveals a multilayered causal structure—"supporting industries → factor conditions → strategic interaction → policy response"—that captures the systemic and cross-domain coupling features of outlet location decisions. Fuzzy DEMATEL effectively detects causal interdependence in small expert samples, complementing SEM-based methods and providing a clear, theoretically sound foundation for strategic decision-making^28,64^.

### Discussion

Based on the fuzzy DEMATEL results, five dimensions and 17 criteria were mapped onto the INRM. By analyzing average centrality (6.216 at the dimension level and 5.338 at the criterion level) and causal degree, the model demonstrates the hierarchical roles and interdependencies among the factors.

#### Dimension-level analysis

Quadrant I (high centrality, high causality): ***R*** is located here, acting as the system’s main driver. Industrial clustering and supply-chain spillovers foster positive interactions among firms, demand-driven strategies, and the government, laying the groundwork for regional competitiveness^8,29^. Enhancing inter-industry connections and supplier networks, therefore, increases both location appeal and systemic efficiency^64^.

Quadrant II (high causality, moderate centrality): ***F*** is located here, representing a stable structural support that ensures long-term viability. Improvements in land, labor, and capital conditions drive market and policy responses, creating a self-reinforcing development cycle^28,53^.

Quadrant III (low centrality, low causality): ***D*** and ***G*** are located here, representing external response factors primarily influenced by market size and the policy environment^10,14^. This pattern corresponds with Porter’s (1998)^22^ idea of a “facilitator government.”

Quadrant IV (high centrality, low causality): ***S*** is located here, representing a highly interactive yet reactive layer that adapts to changes in industrial and resource structures^5,12^.

#### Sub-criterion-level analysis

Quadrant I: Core Driving Factors. ***F2***, ***S1***, ***S3***, ***R3***, and ***G3*** are located here and serve as the system’s primary driving nodes. These indicators influence in multiple directions, and strengthening them can increase the synergetic effect across other areas. Improving ***F2*** can boost land use and market vitality; raising ***S1*** can stimulate demand growth; ***G3*** incentives can attract investment and infrastructure development; and ***R3*** can prolong stay duration and enhance experience stickiness^5,8,29^.

Quadrant II: stable driving factors. ***F1***, ***S4***, ***R1***, and ***R2*** are located here and represent long-term, stable driving forces. ***F1*** investment promotes sustainable development; ***S4*** enhances the customer-attractiveness effect; and ***R1*** and ***R2*** support diverse experiences^28,46^.

Quadrant III: passive response factors. ***D1***, ***D2***, ***D3***, ***G1***, and ***G2*** are located here, representing variables related to external influences. Although they are less controllable, they remain key performance indicators for evaluating the impact of policy and market conditions^10,32^. Improvements in front-end driving sub-criteria, such as ***F2***, ***R3***, and ***G3***, can indirectly improve their performance.

Quadrant IV: key response factors. ***F3***, ***F4***, and ***S2*** are located here, representing influential but weak direct relationships that serve as strategic moderators. ***F3*** and ***F4*** affect accessibility and scalability, while ***S2*** reflects the strength of system feedback^32,58^. These indicators should be monitored regularly to maintain system resilience and support strategic adjustments.

Overall, the outlet mall location system features a three-tier structure—drivers (***R***, ***F***) → mediator (***S***) → responders (***D***, ***G***)—aligned with the causal-layering concept seen in earlier MCDM-based location studies^26,64^. Strengthening key drivers such as ***F2***, ***S1***, ***S3***, ***R3***, and ***G3*** can promote system-wide feedback effects and improve long-term decision stability. Note that this causal structure indicates directional influence rather than decision importance, which is assessed separately using DANP weighting and VIKOR ranking.

### Dimension and sub-criterion weight analysis

To evaluate the significance of each dimension and indicator, this study employed weighted and limit calculations on the total influence matrix obtained from the fuzzy DEMATEL results, as shown in Appendix A, Eqs. (10)–(20) (Table 11). The initial weighted influence matrix for the dimension was then computed (Table 12). Next, the total influence matrix for the sub-criteria was normalized by block and transposed to form the unweighted matrix (Table 13). Then, the weights of the dimensions were multiplied by the sub-criteria’s weighted matrix based on surface size (Table 12 multiplied by Table 13) to generate the sub-criteria’s weighted matrix (Table 14). Finally, through matrix multiplication, the limit matrices for the dimensions and sub-criteria were derived (Tables 15, 16), from which the weights of the dimensions and sub-criteria were identified (Table 17).

**Table 11:** Normalized total influence matrix of dimension.

Dimension	***F***	***D***	***S***	***R***	***G***
*F*	0.145	0.257	0.221	0.162	0.215
*D*	0.202	0.195	0.230	0.158	0.215
*S*	0.197	0.258	0.168	0.167	0.211
*R*	0.195	0.255	0.219	0.123	0.209
*G*	0.183	0.253	0.227	0.177	0.160

**Table 12:** Weighted supermatrix of dimension.

*Dimension*	***F***	***D***	***S***	***R***	***G***
*F*	0.145	0.202	0.197	0.195	0.183
*D*	0.257	0.195	0.258	0.255	0.253
*S*	0.221	0.230	0.168	0.219	0.227
*R*	0.162	0.158	0.167	0.123	0.177
*G*	0.215	0.215	0.211	0.209	0.160

**Table 13:** Unweighted supermatrix of criteria.

*Criteria*	***F1***	***F2***	***F3***	***F4***	***D1***	***D2***	***D3***	***S1***	***S2***	***S3***	***S4***	***R1***	***R2***	***R3***	***G1***	***G2***	***G3***
*F1*	0.126	0.187	0.186	0.174	0.149	0.142	0.152	0.172	0.164	0.172	0.174	0.169	0.171	0.176	0.155	0.149	0.154
*F2*	0.245	0.180	0.243	0.272	0.231	0.234	0.234	0.230	0.225	0.227	0.230	0.224	0.230	0.223	0.235	0.237	0.243
*F3*	0.299	0.301	0.226	0.310	0.291	0.294	0.294	0.286	0.291	0.289	0.283	0.291	0.286	0.288	0.290	0.292	0.287
*F4*	0.329	0.331	0.344	0.244	0.329	0.330	0.320	0.312	0.320	0.312	0.314	0.316	0.312	0.314	0.320	0.322	0.316
*D1*	0.321	0.339	0.341	0.340	0.270	0.382	0.371	0.340	0.340	0.340	0.340	0.341	0.341	0.342	0.341	0.341	0.340
*D2*	0.368	0.359	0.357	0.358	0.398	0.283	0.391	0.357	0.358	0.357	0.357	0.356	0.359	0.359	0.359	0.360	0.358
*D3*	0.311	0.302	0.302	0.301	0.332	0.335	0.239	0.303	0.302	0.304	0.303	0.303	0.300	0.299	0.301	0.299	0.302
*S1*	0.232	0.234	0.230	0.227	0.238	0.229	0.239	0.184	0.255	0.254	0.253	0.233	0.234	0.234	0.246	0.243	0.240
*S2*	0.292	0.292	0.294	0.299	0.291	0.297	0.295	0.316	0.233	0.317	0.309	0.294	0.290	0.290	0.294	0.296	0.284
*S3*	0.231	0.239	0.241	0.244	0.239	0.235	0.239	0.253	0.259	0.186	0.256	0.232	0.233	0.229	0.243	0.245	0.237
*S4*	0.245	0.235	0.234	0.230	0.232	0.238	0.227	0.247	0.253	0.244	0.181	0.241	0.244	0.247	0.216	0.216	0.238
*R1*	0.375	0.374	0.384	0.394	0.376	0.370	0.370	0.360	0.362	0.368	0.373	0.295	0.404	0.402	0.336	0.352	0.365
*R2*	0.307	0.309	0.304	0.302	0.286	0.271	0.288	0.308	0.315	0.310	0.309	0.351	0.238	0.346	0.319	0.313	0.316
*R3*	0.318	0.317	0.312	0.303	0.338	0.360	0.342	0.331	0.323	0.322	0.318	0.354	0.358	0.251	0.345	0.335	0.319
*G1*	0.371	0.369	0.373	0.376	0.372	0.375	0.373	0.330	0.373	0.375	0.374	0.369	0.374	0.372	0.304	0.429	0.391
*G2*	0.395	0.396	0.398	0.403	0.403	0.403	0.400	0.425	0.399	0.399	0.399	0.395	0.395	0.396	0.453	0.329	0.420
*G3*	0.235	0.235	0.230	0.222	0.225	0.222	0.227	0.245	0.228	0.227	0.227	0.236	0.232	0.231	0.243	0.242	0.189

**Table 14:** Weighted supermatrix of criteria.

*Criteria*	*F1*	*F2*	*F3*	*F4*	*D1*	*D2*	*D3*	*S1*	*S2*	*S3*	*S4*	*R1*	*R2*	*R3*	*G1*	*G2*	*G3*
*F1*	0.018	0.027	0.027	0.025	0.030	0.029	0.031	0.034	0.032	0.034	0.034	0.033	0.033	0.034	0.028	0.027	0.028
*F2*	0.036	0.026	0.035	0.039	0.047	0.047	0.047	0.045	0.044	0.045	0.045	0.044	0.045	0.043	0.043	0.043	0.045
*F3*	0.043	0.044	0.033	0.045	0.059	0.059	0.059	0.056	0.057	0.057	0.056	0.057	0.056	0.056	0.053	0.054	0.053
*F4*	0.048	0.048	0.050	0.035	0.066	0.067	0.065	0.061	0.063	0.061	0.062	0.061	0.061	0.061	0.059	0.059	0.058
*D1*	0.083	0.087	0.088	0.088	0.053	0.075	0.072	0.088	0.088	0.088	0.088	0.087	0.087	0.087	0.086	0.086	0.086
*D2*	0.095	0.092	0.092	0.092	0.078	0.055	0.076	0.092	0.092	0.092	0.092	0.091	0.092	0.091	0.091	0.091	0.090
*D3*	0.080	0.078	0.078	0.078	0.065	0.065	0.047	0.078	0.078	0.078	0.078	0.077	0.076	0.076	0.076	0.076	0.076
*S1*	0.051	0.052	0.051	0.050	0.055	0.053	0.055	0.031	0.043	0.043	0.043	0.051	0.051	0.051	0.056	0.055	0.055
*S2*	0.065	0.064	0.065	0.066	0.067	0.068	0.068	0.053	0.039	0.053	0.052	0.064	0.063	0.063	0.067	0.067	0.065
*S3*	0.051	0.053	0.053	0.054	0.055	0.054	0.055	0.043	0.044	0.031	0.043	0.051	0.051	0.050	0.055	0.056	0.054
*S4*	0.054	0.052	0.052	0.051	0.053	0.055	0.052	0.041	0.042	0.041	0.030	0.053	0.053	0.054	0.049	0.049	0.054
*R1*	0.061	0.061	0.062	0.064	0.060	0.058	0.058	0.060	0.060	0.061	0.062	0.036	0.050	0.050	0.059	0.062	0.065
*R2*	0.050	0.050	0.049	0.049	0.045	0.043	0.046	0.051	0.052	0.052	0.052	0.043	0.029	0.043	0.056	0.055	0.056
*R3*	0.052	0.051	0.051	0.049	0.053	0.057	0.054	0.055	0.054	0.054	0.053	0.044	0.044	0.031	0.061	0.059	0.056
*G1*	0.080	0.079	0.080	0.081	0.080	0.080	0.080	0.070	0.079	0.079	0.079	0.077	0.078	0.078	0.049	0.069	0.063
*G2*	0.085	0.085	0.085	0.086	0.086	0.086	0.086	0.090	0.084	0.084	0.084	0.082	0.082	0.083	0.073	0.053	0.067
*G3*	0.050	0.050	0.049	0.048	0.048	0.048	0.049	0.052	0.048	0.048	0.048	0.049	0.048	0.048	0.039	0.039	0.030

**Table 15:** Limiting supermatrix of dimension.

*Dimension*	***F***	***D***	***S***	***R***	***G***
*F*	0.185	0.185	0.185	0.185	0.185
*D*	0.241	0.241	0.241	0.241	0.241
*S*	0.213	0.213	0.213	0.213	0.213
*R*	0.159	0.159	0.159	0.159	0.159
*G*	0.202	0.202	0.202	0.202	0.202

**Table 16:** Limiting supermatrix of criteria.

*Criteria*	***F1***	***F2***	***F3***	***F4***	***D1***	***D2***	***D3***	***S1***	***S2***	***S3***	***S4***	***R1***	***R2***	***R3***	***G1***	***G2***	***G3***
*F1*	0.030	0.030	0.030	0.030	0.030	0.030	0.030	0.030	0.030	0.030	0.030	0.030	0.030	0.030	0.030	0.030	0.030
*F2*	0.043	0.043	0.043	0.043	0.043	0.043	0.043	0.043	0.043	0.043	0.043	0.043	0.043	0.043	0.043	0.043	0.043
*F3*	0.054	0.054	0.054	0.054	0.054	0.054	0.054	0.054	0.054	0.054	0.054	0.054	0.054	0.054	0.054	0.054	0.054
*F4*	0.059	0.059	0.059	0.059	0.059	0.059	0.059	0.059	0.059	0.059	0.059	0.059	0.059	0.059	0.059	0.059	0.059
*D1*	0.082	0.082	0.082	0.082	0.082	0.082	0.082	0.082	0.082	0.082	0.082	0.082	0.082	0.082	0.082	0.082	0.082
*D2*	0.086	0.086	0.086	0.086	0.086	0.086	0.086	0.086	0.086	0.086	0.086	0.086	0.086	0.086	0.086	0.086	0.086
*D3*	0.073	0.073	0.073	0.073	0.073	0.073	0.073	0.073	0.073	0.073	0.073	0.073	0.073	0.073	0.073	0.073	0.073
*S1*	0.050	0.050	0.050	0.050	0.050	0.050	0.050	0.050	0.050	0.050	0.050	0.050	0.050	0.050	0.050	0.050	0.050
*S2*	0.062	0.062	0.062	0.062	0.062	0.062	0.062	0.062	0.062	0.062	0.062	0.062	0.062	0.062	0.062	0.062	0.062
*S3*	0.051	0.051	0.051	0.051	0.051	0.051	0.051	0.051	0.051	0.051	0.051	0.051	0.051	0.051	0.051	0.051	0.051
*S4*	0.049	0.049	0.049	0.049	0.049	0.049	0.049	0.049	0.049	0.049	0.049	0.049	0.049	0.049	0.049	0.049	0.049
*R1*	0.058	0.058	0.058	0.058	0.058	0.058	0.058	0.058	0.058	0.058	0.058	0.058	0.058	0.058	0.058	0.058	0.058
*R2*	0.048	0.048	0.048	0.048	0.048	0.048	0.048	0.048	0.048	0.048	0.048	0.048	0.048	0.048	0.048	0.048	0.048
*R3*	0.052	0.052	0.052	0.052	0.052	0.052	0.052	0.052	0.052	0.052	0.052	0.052	0.052	0.052	0.052	0.052	0.052
*G1*	0.075	0.075	0.075	0.075	0.075	0.075	0.075	0.075	0.075	0.075	0.075	0.075	0.075	0.075	0.075	0.075	0.075
*G2*	0.081	0.081	0.081	0.081	0.081	0.081	0.081	0.081	0.081	0.081	0.081	0.081	0.081	0.081	0.081	0.081	0.081
*G3*	0.046	0.046	0.046	0.046	0.046	0.046	0.046	0.046	0.046	0.046	0.046	0.046	0.046	0.046	0.046	0.046	0.046

**Table 17:** Fuzzy DANP weighting of dimensions and criteria.

*Dimension*	Limit weight	Rank	*Criteria*	Limit weight	Rank
Factors of production (***F***)	0.185	4	Capital Investment (***F1***)	0.030	17
			Labor Conditions (***F2***)	0.043	15
			Land Area (***F3***)	0.054	8
			Site Visibility (***F4***)	0.059	6
Demand conditions (***D***)	0.241	1	Purchasing Power (**D1**)	0.082	2
			Population Size (***D2***)	0.086	1
			Customer Demographics (***D3***)	0.073	5
Firm strategy, structure, and rivalry (***S***)	0.213	2	Brand Visibility (***S1***)	0.050	12
			Market Growth Potential (***S2***)	0.062	7
			Rivalry (***S3***)	0.051	11
			Business District Compatibility (***S4***)	0.049	13
Related and supporting industries (***R***)	0.159	5	Boutique Suppliers (***R1***)	0.058	9
			Entertainment Suppliers (***R2***)	0.048	14
			Food Court Suppliers (***R3***)	0.052	10
Government(***G***)	0.202	3	Regional Development (***G1***)	0.075	4
			Traffic Conditions (***G2***)	0.081	3
			Regulations and Incentives (***G3***)	0.046	16

The DANP analysis results reveal that dimension ***D*** has the highest weight (0.241), followed by ***S*** (0.213) and ***G*** (0.202), indicating that market structure, brand strategy, and policy stability are the three key pillars of decision-making. ***F*** and ***R*** are driving dimensions; their weights are slightly lower (0.185 and 0.159, respectively) because their feedback mechanisms are relatively limited, representing indirect factors that influence the competitive advantage of outlet malls.

At the sub-criteria level, ***D2*** and ***D1*** are the two indicators with the highest weights (0.086 and 0.082, respectively), indicating the dominant influence of market potential and consumption structure on site selection decisions. ***G2*** and ***G1*** are next, reflecting the key role of location accessibility and infrastructure. In contrast, ***F1*** and ***F2*** have lower weights; although they have high driving forces, they mainly provide basic support and have low marginal benefits.

From a weight-focused perspective, the decision emphasis in outlet site selection is mainly driven by demand-related indicators, transportation accessibility, and policy support, aligning with Porter’s (1998a, 1998b) theory of competitive advantage^22,23^. This weight-based prioritization does not contradict the causal structure identified by fuzzy DEMATEL; instead, it reflects performance-oriented considerations that influence the final ranking stage.

### VIKOR ranking and sensitivity analysis

Based on the weights for dimensions and sub-criteria obtained from the fuzzy DEMATEL–DANP framework, this study further used the VIKOR method to evaluate and rank three candidate outlet locations: Yongkang, Rende, and Guiren. The expert panel provided quantitative assessments of 17 evaluation criteria (Table 18), reflecting perceived relative advantages rather than objective costs or resource-consumption measures. Using Eqs. (1),(2), the distances between each alternative and the ideal solution were calculated. The results show that Guiren District (***Q*** = 0.000) is the preferred location, followed by Yongkang District (***Q*** = 0.489) and Rende District (***Q*** = 1.000) (Table 19).

**Table 18:** Preliminary expert ratings for the three alternative options.

Alternatives	*Criteria*																
	***F1***	***F2***	***F3***	***F4***	***D1***	***D2***	***D3***	***S1***	***S2***	***S3***	***S4***	***R1***	***R2***	***R3***	***G1***	***G2***	***G3***
A (Yongkang District)	7.00	8.00	7.00	9.00	9.50	9.00	8.50	9.00	7.50	8.00	8.50	8.50	8.00	8.00	8.00	7.50	8.00
B (Guiren District)	8.50	8.00	9.50	8.50	9.00	8.50	8.00	8.50	9.00	9.00	8.50	8.00	7.00	7.00	8.50	9.00	9.00
C (Rende District)	7.50	7.50	9.00	7.50	8.50	6.50	7.50	8.00	7.00	8.00	6.50	8.00	6.00	6.00	7.50	7.00	7.00

**Table 19:** Resultant of $U$, $V,$ and ${\boldsymbol{Q}}$.

Alternatives	*Separation measure*			
	$U$	$V$	${\boldsymbol{Q}}$	Rank
A (Yongkang District)	0.164	0.164	0.489	2
B (Guiren District)	0.092	0.092	0.000	1
C (Rende District)	0.240	0.240	1.000	3

Guiren District performs strongly in both the group utility index ($U$) and the individual regret index ($V$), mainly because of its advantages in land availability, transportation access, and policy incentives, including plenty of developable land, a vast transportation network, and supportive government measures. Yongkang District, despite its high population density and strong consumption potential, is limited by land shortages, higher development costs, and intense market competition. Rende District ranks lowest, primarily due to weaker market activity and less transportation connectivity.

To evaluate the stability of the VIKOR ranking under different decision-making preferences, a sensitivity analysis was further performed. It should be emphasized that this analysis is not intended to reflect the most likely real-world weight configurations but rather to serve as a preference-driven stress test. Specifically, it investigates how the ranking of alternative locations might change when decision-makers prioritize specific dimensions or criteria, thereby highlighting factors that have a critical impact on decision outcomes.

**Fig. 3:**
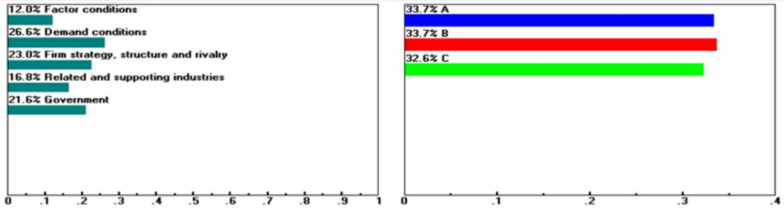
Sensitivity of the dynamics of the following nodes: Goal when “Demand Conditions” increases from 24.2 to 26.6%.

**Fig. 4:**
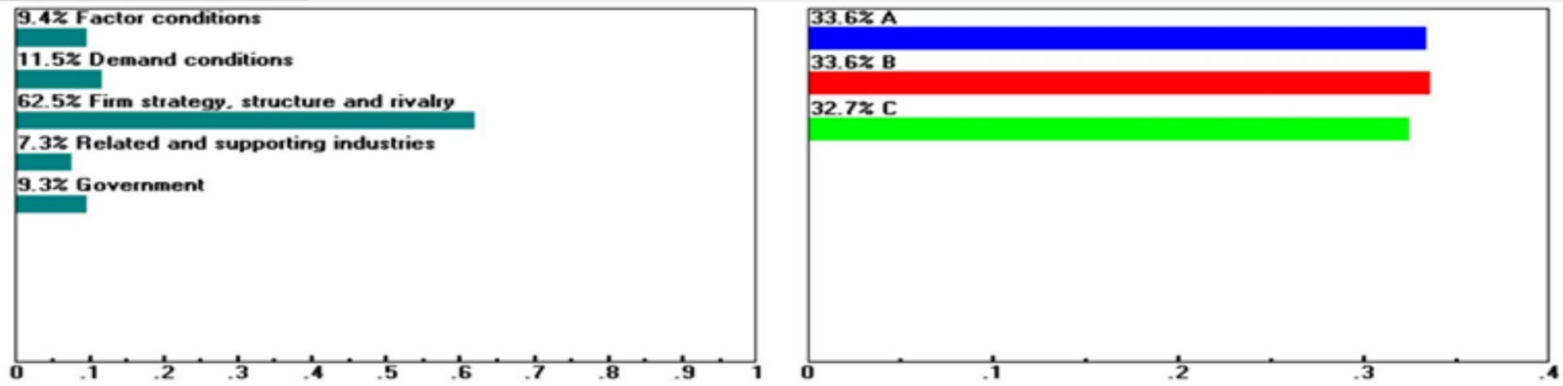
Sensitivity of the dynamics of the following nodes: Goal when "Firm Strategy, Structure and Rivalry" increases from 21.0 to 62.5%.

Using expert choice (version 11.5, Expert Choice Inc., USA), systematic perturbations were applied to the original weighting structure derived from DANP analysis. Under the baseline weight configuration, the ranking of alternatives remains Guiren District (B), followed by Yongkang District (A), and Rende District (C). When the weight of the Demand Conditions dimension increases from 24.2 to 26.6%, or when the Firm Strategy, Structure, and Rivalry dimension is strongly emphasized, Yongkang District may surpass Guiren District in the ranking (see Figs. 3, 4). This finding suggests that Yongkang District has latent advantages in these areas, although their realization heavily depends on decision-makers’ preference orientations.

**Fig. 5:**
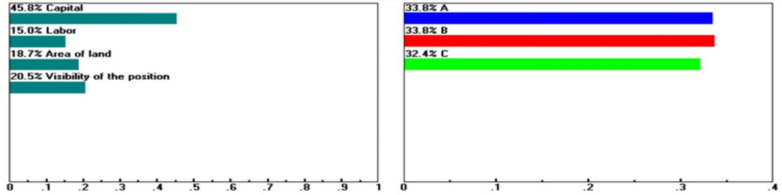
Sensitivity of the dynamics of the following nodes: Goal > "Factors of Production" (L: 0.199) when “Capital” increases from 16.5 to 45.8%.

**Fig. 6:**
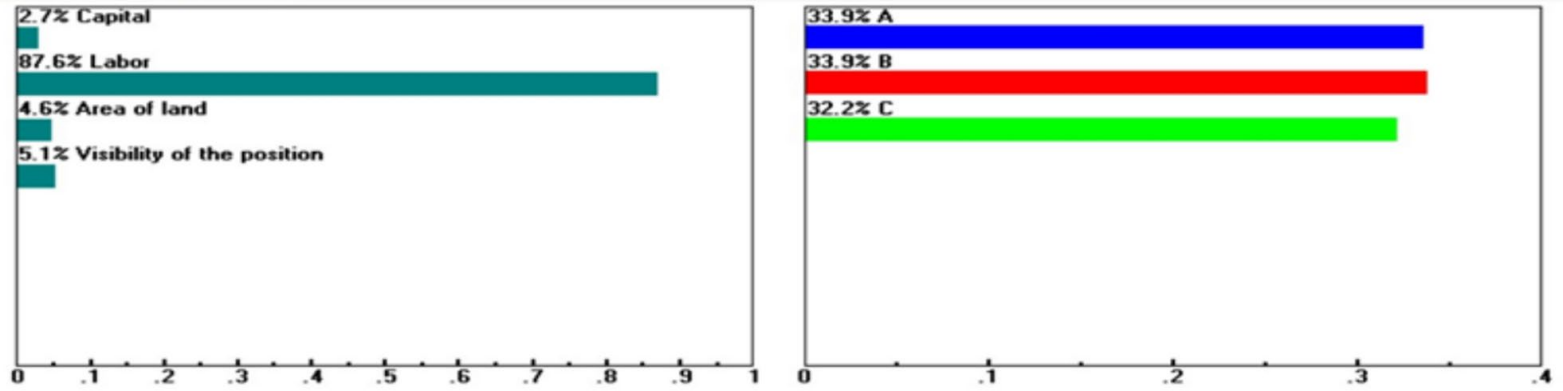
Sensitivity of the dynamics of below nodes: Goal > "Factors of Production" (L: 0.199) when “Labor” increases from 23.1 to 87.6%.

**Fig. 7:**
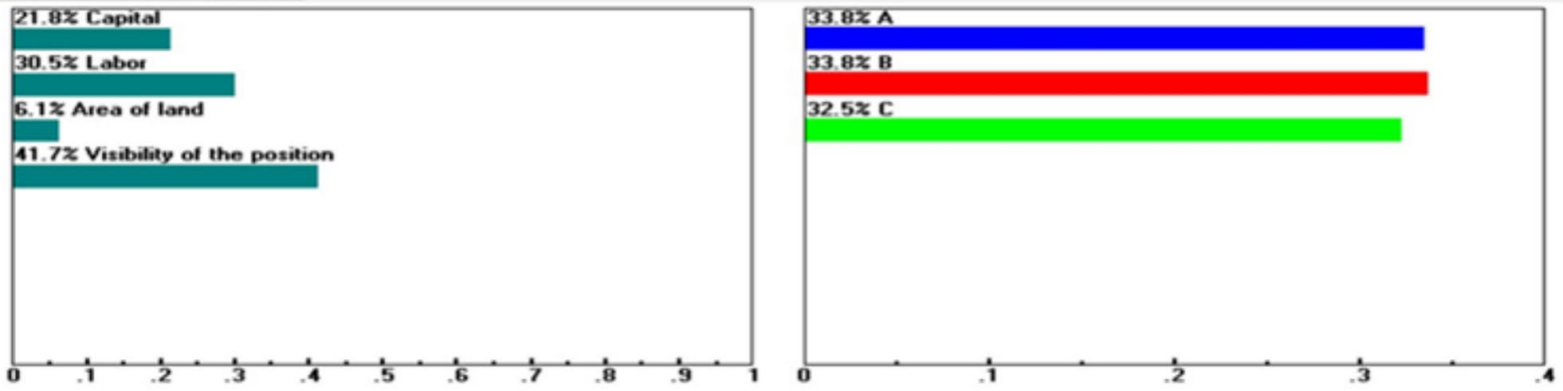
Sensitivity of the dynamics of the following nodes: Goal > "Factors of Production" (L: 0.199) when “Site Visibility” increases from 31.6 to 41.7%.

**Fig. 8:**
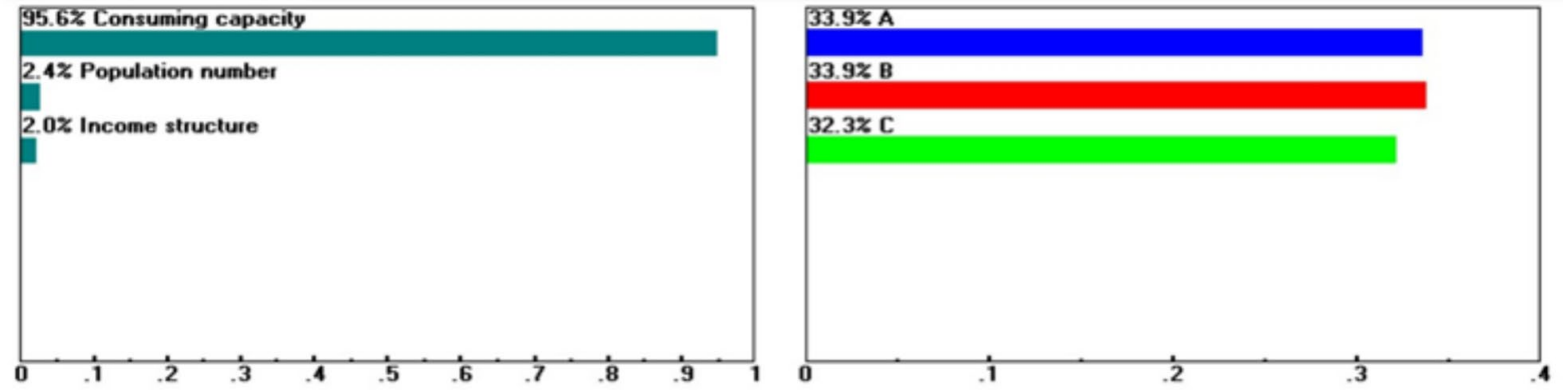
Sensitivity of the dynamics of the following nodes: Goal > “Demand Conditions” (L: 0.242) when “Purchasing Power” increases from 33.9 to 95.6%.

Within the Factor Conditions dimension, Guiren District remains the preferred choice when the baseline weights for capital investment (16.5%), labor conditions (23.1%), site visibility (31.6%), and purchasing power (33.9%) are maintained. However, when these criteria are assigned extreme weights of 45.8, 87.6, 41.7, and 95.6%, respectively, as boundary-testing scenarios rather than realistic configurations (see Figs. 5, 6, 7, 8), Yongkang District may rank first. These scenarios do not suggest the practical feasibility of these weight settings in real-world decision-making but rather illustrate which dimensions or criteria are more sensitive and exert greater influence when decision-makers’ preferences shift significantly.

Overall, the sensitivity analysis shows that the VIKOR ranking remains relatively consistent across reasonable weight ranges and changes only when there is a strong preference bias toward specific dimensions or criteria. From a decision-support perspective, these results help identify location options that are structurally solid versus those that are more sensitive to preferences, thereby improving the clarity and practical usefulness of the proposed decision framework^19,28^.

### Managerial implications

Based on the combined results of fuzzy DEMATEL, DANP, and VIKOR, this study offers the following strategic recommendations, focusing on sub-criteria-level optimization.

1. Industry Support Strategy: Centered on ***R3***, strengthens the synergistic effect between catering, entertainment, and retail to enhance experience engagement and location attractiveness.
2. Factor Condition Strategy: Focus on long-term, stable investments in ***F2*** and ***F1***, improve talent development and the funding environment, and ensure resilience in growth.
3. Strategic and Competitive Strategy: Improve monitoring systems for ***S1*** and ***S3*** to foster brand differentiation and market regulation mechanisms.
4. Policy-Focused Strategy: Use ***G3*** to guide investment through tax incentives and land support, encouraging joint development between government and businesses.

In summary, outlet site selection should consider both driving structure and weight orientation together. The driving factors identified by fuzzy DEMATEL show where strategic actions can produce system-wide effects, while high-weight factors from DANP–VIKOR serve as performance targets that help compare sites in a changing market environment.

## Conclusions and limitations

This study develops a theory-informed, expert-based decision-support framework for outlet mall location planning by combining a hybrid MCDM structure within Porter’s Diamond Model. Instead of predicting optimal outcomes or testing causal hypotheses, the framework aims to clarify structural influence patterns and preference-sensitive relationships among location-related factors during the pre-investment phase.

The results show that Factor Conditions and Related and Supporting Industries serve as structurally influential components of the decision network. Meanwhile, Firm Strategy, Structure, and Rivalry mainly play a mediating and feedback role. Demand Conditions and Government Roles function primarily as outcome-oriented dimensions with higher decision weights. At the sub-criteria level, labor conditions, experiential service supply, brand visibility, market growth potential, and regulatory incentives have a more substantial structural influence. In contrast, purchasing power, population-related demand indicators, and transportation accessibility dominate weight-based evaluations. These findings suggest that outlet mall location competitiveness stems from the interaction of industrial structure, strategic positioning, and policy context, rather than any single dominant factor. Accordingly, causal influence, criterion importance, and ranking performance are distinct yet complementary outputs of the proposed framework.

Sensitivity analysis further shows that the VIKOR ranking remains consistent across reasonable weight changes and shifts primarily when decision-makers heavily emphasize specific dimensions or criteria. This preference-based stress testing helps distinguish locations with inherently substantial advantages from those whose competitiveness relies on particular strategic or policy assumptions, making expert-based rankings more understandable in high-capital investment decisions.

Several limitations should be acknowledged. The expert-based evaluation depends on a relatively small panel. It examines only three candidate locations within a single regional context, reflecting the realistically constrained pre-investment decision setting rather than an arbitrary research design. Nonetheless, this restricts statistical generalization and the assessment of discriminatory power across larger choice sets. Additionally, the framework offers a static, perception-based evaluation and does not incorporate temporal dynamics or validate its performance using observed data. Future research could expand this approach by integrating objective indicators, longitudinal data, or hybrid expert-data models, and by applying the framework to larger or cross-regional candidate pools to assess its robustness and stability further.

## Data Availability

The datasets generated during and/or analyzed in the current study are not publicly available due to institutional privacy policies and the ongoing ethical review at China Medical University. However, they are available from the corresponding author, Jhong-Min Yang (email: t2026003@gm.cyut.edu.tw), upon reasonable request.

## Appendix A: Fuzzy DEMATEL approach

### Step 1: Examination of consistency

This step mainly checks whether the questionnaire responses from the ***k*** experts reach a consensus. Therefore, Eq. (1) will be used for the consistency test. When the consistency ratio is less than the significance level (usually set at α = 5%), the questionnaire responses from k experts are considered consistent and indicate consensus. Otherwise, the experts need to resubmit their responses.

A1 $$Consistency check{ = }\frac{1}{{n\left( {n - 1} \right)}}\sum\limits_{i = 1}^{n} {\sum\limits_{j = 1}^{n} {\left( {{{\left| {\overline{{\boldsymbol{\alpha}}}_{ij}^{k} - \overline{{\boldsymbol{\alpha}}}_{ij}^{k - 1} } \right|} \mathord{\left/ {\vphantom {{\left| {\overline{{\boldsymbol{\alpha}}}_{ij}^{k} - \overline{{\boldsymbol{\alpha}}}_{ij}^{k - 1} } \right|} {\overline{{\boldsymbol{\alpha}}}_{ij}^{k} }}} \right. \kern-0pt} {\overline{{\boldsymbol{\alpha}}}_{ij}^{k} }}} \right)} } \times 100\% < 5\% ,$$

where ***n*** represents the number of criteria, and ***k*** represents the number of experts.

*Step* 2*. Summarize the expert evaluation results and calculate the average initial fuzzy relationship matrix*.

Let ${\boldsymbol{a}}_{{{\boldsymbol{ij}}}}^{{\boldsymbol{k}}} = ({\boldsymbol{al}}_{ij}^{{\boldsymbol{k}}} ,{\boldsymbol{am}}_{ij}^{{\boldsymbol{k}}} ,{\boldsymbol{ar}}_{ij}^{{\boldsymbol{k}}} )$, having ${\boldsymbol{k}}$ experts, fuzzy weight of $am_{{{\boldsymbol{ij}}}}^{{\boldsymbol{k}}}$ denotes the $i^{th}$ criteria, and it affects the $j^{th}$ criteria to be evaluated by ${\boldsymbol{k}}^{th}$ experts.

(1) Normalization:

A2 $$\begin{array}{*{20}c} {{\boldsymbol{\varDelta}}_{\min }^{\max } = \max {\boldsymbol{ar}}_{ij}^{{\boldsymbol{k}}} - {\boldsymbol{al}}_{ij}^{{\boldsymbol{k}}} \quad \quad \;\;\;} \\ {{\boldsymbol{xal}}_{ij}^{{\boldsymbol{k}}} = ({\boldsymbol{al}}_{ij}^{{\boldsymbol{k}}} - \min {\boldsymbol{al}}_{ij}^{{\boldsymbol{k}}} )/{\boldsymbol{\varDelta}}_{\min }^{\max } } \\ {{\boldsymbol{xam}}_{ij}^{{\boldsymbol{k}}} = ({\boldsymbol{am}}_{ij}^{{\boldsymbol{k}}} - \min {\boldsymbol{al}}_{ij}^{{\boldsymbol{k}}} )/{\boldsymbol{\varDelta}}_{\min }^{\max } } \\ {{\boldsymbol{xar}}_{ij}^{{\boldsymbol{k}}} = ({\boldsymbol{ar}}_{ij}^{{\boldsymbol{k}}} - \min {\boldsymbol{al}}_{ij}^{{\boldsymbol{k}}} )/{\boldsymbol{\varDelta}}_{\min }^{\max } } \\ \end{array}$$

(2) Calculate the normalized values of left (***ls***) and right (***rs***):

A3 $$\begin{array}{*{20}c} {{\boldsymbol{xls}}_{{\boldsymbol{j}}}^{{\boldsymbol{k}}} = {{{\boldsymbol{xam}}_{{{\boldsymbol{ij}}}}^{{\boldsymbol{k}}} } \mathord{\left/ {\vphantom {{{\boldsymbol{xam}}_{{{\boldsymbol{ij}}}}^{{\boldsymbol{k}}} } {(1 + {\boldsymbol{xam}}_{{{\boldsymbol{ij}}}}^{{\boldsymbol{k}}} - {\boldsymbol{xal}}_{{{\boldsymbol{ij}}}}^{{\boldsymbol{k}}} )}}} \right. \kern-0pt} {(1 + {\boldsymbol{xam}}_{{{\boldsymbol{ij}}}}^{{\boldsymbol{k}}} - {\boldsymbol{xal}}_{{{\boldsymbol{ij}}}}^{{\boldsymbol{k}}} )}}} \\ {{\boldsymbol{xrs}}_{{\boldsymbol{j}}}^{{\boldsymbol{k}}} = {{{\boldsymbol{xar}}_{{{\boldsymbol{ij}}}}^{{\boldsymbol{k}}} } \mathord{\left/ {\vphantom {{{\boldsymbol{xar}}_{{{\boldsymbol{ij}}}}^{{\boldsymbol{k}}} } {(1 + {\boldsymbol{xar}}_{{{\boldsymbol{ij}}}}^{{\boldsymbol{k}}} - {\boldsymbol{xam}}_{{{\boldsymbol{ij}}}}^{{\boldsymbol{k}}} )}}} \right. \kern-0pt} {(1 + {\boldsymbol{xar}}_{{{\boldsymbol{ij}}}}^{{\boldsymbol{k}}} - {\boldsymbol{xam}}_{{{\boldsymbol{ij}}}}^{{\boldsymbol{k}}} )}}} \\ \end{array}$$

(3) Calculate the overall normalized crisp value:

A4 $${\boldsymbol{x}}_{j}^{{crisp^{k} }} = {{[{\boldsymbol{xls}}_{ij}^{k} (1 - {\boldsymbol{xls}}_{ij}^{k} ) + {\boldsymbol{xrs}}_{ij}^{k} \cdot {\boldsymbol{xrs}}_{ij}^{k} ]} \mathord{\left/ {\vphantom {{[{\boldsymbol{xls}}_{ij}^{k} (1 - {\boldsymbol{xls}}_{ij}^{k} ) + {\boldsymbol{xrs}}_{ij}^{k} \cdot {\boldsymbol{xrs}}_{ij}^{k} ]} {[1 - {\boldsymbol{xls}}_{ij}^{k} }}} \right. \kern-0pt} {[1 - {\boldsymbol{xls}}_{ij}^{k} }} + {\boldsymbol{xrs}}_{ij}^{k} ]$$

(4) Calculation of crisp values for each expert questionnaire:

A5 $${\boldsymbol{h}}_{{{\boldsymbol{ij}}}}^{{\boldsymbol{k}}} = \min {\boldsymbol{al}}_{{{\boldsymbol{ij}}}}^{{\boldsymbol{k}}} + {\boldsymbol{x}}_{{\boldsymbol{j}}}^{{{\boldsymbol{crisp}}^{{\boldsymbol{k}}} }} \cdot{\boldsymbol{\varDelta}}_{\min }^{\max }$$

(5) Integration of crisp values from all expert questionnaires:

A6 $$b_{ij} = \frac{1}{{\boldsymbol{k}}}(h_{ij}^{1} + h_{ij}^{2} + \cdots + h_{ij}^{{\boldsymbol{k}}} )$$

The value of the fuzzy direct correlation matrix $B = [ \, b_{ij} ]_{n \times n}$ is derived from the average of the same criteria across the various fuzzy direct matrices via equations (A2)-(A6) for all questionnaire respondents.

*Step* 3: *Calculate the total impact matrix*.

Normalize the mean matrix $B$ to obtain the initial inflect matrix $E = [ \, e_{ij} ]_{n \times n}$, that is, the matrix $E$ can be obtained through equations (A7) to (A8), in which all principal diagonal elements equal zero.

A7 $$E = B\user2{/}\kappa$$

A8 $$\kappa = \min \left[ {\mathop {\max }\limits_{i} \sum\nolimits_{{j = 1}}^{n} {|b_{{ij}} |} ,\mathop {\max }\limits_{j} \sum\nolimits_{{i = 1}}^{n} {|b_{{ij}} |} } \right]$$

The direct relationship matrix E yields the total influence matrix ${\boldsymbol{T}}$ by equation (A8), that is,

A9 $$T = \left[ {t_{ij} } \right]_{n \times n} = E + E^{2} + E^{3} + \cdots + E^{\infty } \approx E(I - E)^{ - 1}$$

where*** I*** am indicated as the identity matrix and $\;E^{\infty } \approx \left[ 0 \right]_{n \times n}$.

*Step* 4: *The Influential Network Relationship Map (INRM) is obtained from expert comments by setting a threshold value*.

The variables ${\boldsymbol{K}}$ and ${\boldsymbol{H}}$ are as follows.

A10 $$K = \left[ {k_{j} } \right]_{n \times 1} = \left[ {\sum\nolimits_{i = 1}^{n} {t_{ij} } } \right]^{T}_{1 \times n} ,\;\;H = \left[ {h_{i} } \right]_{n \times 1} = \left[ {\sum\nolimits_{j = 1}^{n} {t_{ij} } } \right]_{n \times 1} .$$

where the threshold value is obtained by calculating the average of the total influence matrix ${\boldsymbol{T}}$. ${\boldsymbol{H}}{ + }{\boldsymbol{K}}$ indicates the relative importance of each factor, while ${\boldsymbol{H}}{ - }{\boldsymbol{K}}$ reflects the causal relationship between the factors.

*Step* 5*: Dimension-level weight matrix and limit matrix.*

To analyze the relative influence among primary criteria (dimensions), the initial relation matrix for each dimension is first processed with DEMATEL to produce the total influence matrix at the dimension level. This matrix reveals the combined causal relationships among the dimensions. Next, the matrix is normalized and transposed to create the dimension-level weighting matrix, as shown in Equations (A11) to (A13). To ensure stability and consistency in the weighting structure, the normalized matrix is repeatedly multiplied until it converges to the dimension-level matrix, as shown in equation (A14).

A11 $$\begin{array}{*{20}c} {{\boldsymbol{T}}_{dim} } & {\begin{array}{*{20}c} = & {\left[ {\begin{array}{*{20}c} {t_{11}^{dim} } & \cdots & {t_{1j}^{dim} } & \cdots & {t_{1m}^{dim} } \\ \vdots & {} & \vdots & {} & \vdots \\ {t_{i1}^{dim} } & \cdots & {t_{ij}^{dim} } & \cdots & {t_{im}^{dim} } \\ \vdots & {} & \vdots & {} & \vdots \\ {t_{m1}^{dim} } & \cdots & {t_{mj}^{dim} } & \cdots & {t_{mm}^{dim} } \\ \end{array} } \right]} \\ \end{array} } \\ \end{array} \begin{array}{*{20}c} \to \\ {} \\ \to \\ {} \\ \to \\ \end{array} \begin{array}{*{20}c} {\omega_{1} = \sum\nolimits_{j = 1}^{m} {t_{1j}^{dim} } } \\ \vdots \\ {\omega_{i} = \sum\nolimits_{j = 1}^{m} {t_{ij}^{dim} } } \\ \vdots \\ {\omega_{m} = \sum\nolimits_{j = 1}^{m} {t_{mj}^{dim} } } \\ \end{array}$$

A12 $$T_{{_{\dim } }}^{*} = \left[ {\begin{array}{*{20}c} {{{t_{11}^{{_{\dim } }} } \mathord{\left/ {\vphantom {{t_{11}^{{_{\dim } }} } {\omega_{1} }}} \right. \kern-0pt} {\omega_{1} }}} & \cdots & {{{t_{1j}^{{_{\dim } }} } \mathord{\left/ {\vphantom {{t_{1j}^{{_{\dim } }} } {\omega_{1} }}} \right. \kern-0pt} {\omega_{1} }}} & \cdots & {{{t_{1m}^{{_{\dim } }} } \mathord{\left/ {\vphantom {{t_{1m}^{{_{\dim } }} } {\omega_{1} }}} \right. \kern-0pt} {\omega_{1} }}} \\ \vdots & {} & \vdots & {} & \vdots \\ {{{t_{i1}^{{_{\dim } }} } \mathord{\left/ {\vphantom {{t_{i1}^{{_{\dim } }} } {\omega_{i} }}} \right. \kern-0pt} {\omega_{i} }}} & \cdots & {{{t_{ij}^{{_{\dim } }} } \mathord{\left/ {\vphantom {{t_{ij}^{{_{\dim } }} } {\omega_{i} }}} \right. \kern-0pt} {\omega_{i} }}} & \cdots & {{{t_{im}^{{_{\dim } }} } \mathord{\left/ {\vphantom {{t_{im}^{{_{\dim } }} } {\omega_{i} }}} \right. \kern-0pt} {\omega_{i} }}} \\ \vdots & {} & \vdots & {} & \vdots \\ {{{t_{m1}^{{_{\dim } }} } \mathord{\left/ {\vphantom {{t_{m1}^{{_{\dim } }} } {\omega_{m} }}} \right. \kern-0pt} {\omega_{m} }}} & \cdots & {{{t_{mj}^{{_{\dim } }} } \mathord{\left/ {\vphantom {{t_{mj}^{{_{\dim } }} } {\omega_{m} }}} \right. \kern-0pt} {\omega_{m} }}} & \cdots & {{{t_{mm}^{{_{\dim } }} } \mathord{\left/ {\vphantom {{t_{mm}^{{_{\dim } }} } {\omega_{m} }}} \right. \kern-0pt} {\omega_{m} }}} \\ \end{array} } \right] = \left[ {\begin{array}{*{20}c} {t_{11}^{{_{\dim }^{*} }} } & \cdots & {t_{1j}^{{_{\dim }^{*} }} } & \cdots & {t_{1m}^{{_{\dim }^{*} }} } \\ \vdots & {} & \vdots & {} & \vdots \\ {t_{i1}^{{_{\dim }^{*} }} } & \cdots & {t_{ij}^{{_{\dim }^{*} }} } & \cdots & {t_{im}^{{_{\dim }^{*} }} } \\ \vdots & {} & \vdots & {} & \vdots \\ {t_{m1}^{{_{\dim }^{*} }} } & \cdots & {t_{mj}^{{_{\dim }^{*} }} } & \cdots & {t_{mm}^{{_{\dim }^{*} }} } \\ \end{array} } \right]\begin{array}{*{20}c} \to \\ {} \\ \to \\ {} \\ \to \\ \end{array} \begin{array}{*{20}c} {\sum\nolimits_{j = 1}^{m} {t_{1j}^{{_{\dim }^{*} }} = 1} } \\ \vdots \\ {\sum\nolimits_{j = 1}^{m} {t_{ij}^{{_{\dim }^{*} }} = 1} } \\ \vdots \\ {\sum\nolimits_{j = 1}^{m} {t_{mj}^{{_{\dim }^{*} }} = 1} } \\ \end{array}$$

A13 $$T_{{_{\dim } }}^{w} = \left[ {T_{{_{\dim } }}^{*} } \right]^{T} = \left[ {\begin{array}{*{20}c} {t_{11}^{{_{\dim }^{*} }} } & \cdots & {t_{i1}^{{_{\dim }^{*} }} } & \cdots & {t_{m1}^{{_{\dim }^{*} }} } \\ \vdots & {} & \vdots & {} & \vdots \\ {t_{1j}^{{_{\dim }^{*} }} } & \cdots & {t_{ij}^{{_{\dim }^{*} }} } & \cdots & {t_{mj}^{{_{\dim }^{*} }} } \\ \vdots & {} & \vdots & {} & \vdots \\ {t_{1m}^{{_{\dim }^{*} }} } & \cdots & {t_{im}^{{_{\dim }^{*} }} } & \cdots & {t_{mm}^{{_{\dim }^{*} }} } \\ \end{array} } \right]$$

A14 $${\boldsymbol{T}}_{{_{dim} }}^{lim} = \mathop {\lim }\limits_{k \to \infty } \left[ {{\boldsymbol{T}}_{{_{dim} }}^{w} } \right]^{k}$$

The limit matrix (equation A14) shows the stable weights for each dimension, including both direct and indirect influences within the semantic network.

*Step* 6*: Constructing the supermatrix for criteria-level weighting.*

First, the overall relationship matrix of sub-criteria is divided into sub-matrices based on the size of their dimensional structures. For example, with ***m*** criteria (dimensions), each containing ***n*** sub-criteria, the total relation matrix of the sub-criteria consists of ***m***** × *****n*** submatrices, as shown in equation (A15). These submatrices illustrate how indicators interact within and across dimensions. After division, each submatrix is normalized using the dimensional normalization steps (see Equations A11-A13 to ensure proportional influence, preserve the network’s logical structure, and align with the dimensional weighting scheme. Note that after normalization, the sum of each row is m. This process helps properly adjust the influence of the criteria. Finally, the total relation matrix is transposed to form the unweighted supermatrix of the criteria (see equations A15–A19).

A15 $$\quad {\boldsymbol{T}}_{c}^{{}} = \begin{array}{*{20}c} {} & {} & {\begin{array}{*{20}c} {\begin{array}{*{20}c} {D_{1} } \\ {C_{11} \cdots C_{{1m_{1} }} } \\ \end{array} } & {\begin{array}{*{20}c} {} \\ \cdots \\ \end{array} } & {\begin{array}{*{20}c} {D_{j} } \\ {C_{j1} \cdots C_{{jm_{j} }} } \\ \end{array} } & {\begin{array}{*{20}c} {} \\ \cdots \\ \end{array} } & {\begin{array}{*{20}c} {D_{m} } \\ {C_{m1} \cdots C_{{mm_{n} }} } \\ \end{array} } \\ \end{array} } \\ {\begin{array}{*{20}c} {\begin{array}{*{20}c} {} \\ {D_{1} } \\ {} \\ \end{array} } \\ \vdots \\ {\begin{array}{*{20}c} {} \\ {D_{i} } \\ {} \\ \end{array} } \\ \vdots \\ {\begin{array}{*{20}c} {} \\ {D_{m} } \\ {} \\ \end{array} } \\ \end{array} } & {\begin{array}{*{20}c} {\begin{array}{*{20}c} {C_{11} } \\ \vdots \\ {C_{{1m_{1} }} } \\ \end{array} } \\ \vdots \\ {\begin{array}{*{20}c} {C_{i1} } \\ \vdots \\ {C_{{im_{i} }} } \\ \end{array} } \\ \vdots \\ {\begin{array}{*{20}c} {C_{m1} } \\ \vdots \\ {C_{{mm_{n} }} } \\ \end{array} } \\ \end{array} } & {\left[ {\begin{array}{*{20}c} {{\boldsymbol{T}}_{c}^{\beta 11} } & \cdots & {{\boldsymbol{T}}_{c}^{{\beta_{1} j}} } & \cdots & {{\boldsymbol{T}}_{c}^{{\beta_{1} m}} } \\ \vdots & {} & \vdots & {} & \vdots \\ {{\boldsymbol{T}}_{c}^{\beta i1} } & \cdots & {{\boldsymbol{T}}_{c}^{\beta ij} } & \cdots & {{\boldsymbol{T}}_{c}^{\beta im} } \\ \vdots & {} & \vdots & {} & \vdots \\ {{\boldsymbol{T}}_{c}^{\beta m1} } & \cdots & {{\boldsymbol{T}}_{c}^{\beta mj} } & \cdots & {{\boldsymbol{T}}_{c}^{\beta mm} } \\ \end{array} } \right]} \\ \end{array} \;{ = }\left[ {\begin{array}{*{20}c} {{\boldsymbol{T}}_{11} } & \vdots & {{\boldsymbol{T}}_{1j} } & \vdots & {{\boldsymbol{T}}_{1m} } \\ \cdots & \vdots & \cdots & \vdots & \cdots \\ {{\boldsymbol{T}}_{i1} } & \vdots & {{\boldsymbol{T}}_{ij} } & \vdots & {{\boldsymbol{T}}_{im} } \\ \cdots & \vdots & \cdots & \vdots & \cdots \\ {{\boldsymbol{T}}_{m1} } & \vdots & {{\boldsymbol{T}}_{mj} } & \vdots & {{\boldsymbol{T}}_{mm} } \\ \end{array} } \right],$$

A16 $$\begin{array}{*{20}c} {{\boldsymbol{T}}_{11} } & {\begin{array}{*{20}c} = & {\left[ {\begin{array}{*{20}c} {{\boldsymbol{t}}_{11}^{{}} } & \cdots & {{\boldsymbol{t}}_{1j}^{{}} } & \cdots & {{\boldsymbol{t}}_{{1n_{1} }}^{{}} } \\ \vdots & {} & \vdots & {} & \vdots \\ {{\boldsymbol{t}}_{i1}^{{}} } & \cdots & {{\boldsymbol{t}}_{ij}^{{}} } & \cdots & {{\boldsymbol{t}}_{{in_{1} }}^{{}} } \\ \vdots & {} & \vdots & {} & \vdots \\ {{\boldsymbol{t}}_{{n_{1} 1}}^{{}} } & \cdots & {{\boldsymbol{t}}_{{n_{1} j}}^{{}} } & \cdots & {{\boldsymbol{t}}_{{{}_{{n_{1} n_{1} }}}}^{{}} } \\ \end{array} } \right]} \\ \end{array} } \\ \end{array} \begin{array}{*{20}c} \to \\ {} \\ \to \\ {} \\ \to \\ \end{array} \begin{array}{*{20}c} {\varpi_{1}^{11} = \sum\nolimits_{j = 1}^{{n_{1} }} {{\boldsymbol{t}}_{1j}^{{}} } } \\ \vdots \\ {\varpi_{i}^{11} = \sum\nolimits_{j = 1}^{{n_{1} }} {{\boldsymbol{t}}_{ij}^{{}} } } \\ \vdots \\ {\varpi_{{_{{n_{1} }} }}^{11} = \sum\nolimits_{j = 1}^{{n_{1} }} {{\boldsymbol{t}}_{{n_{1} j}}^{{}} } } \\ \end{array}$$

A17 $$\begin{array}{*{20}c} {{\boldsymbol{T}}_{11}^{*} } & {\begin{array}{*{20}c} = & {\left[ {\begin{array}{*{20}c} {{{{\boldsymbol{t}}_{11}^{{}} } \mathord{\left/ {\vphantom {{{\boldsymbol{t}}_{11}^{{}} } {\varpi_{1}^{11} }}} \right. \kern-0pt} {\varpi_{1}^{11} }}} & \cdots & {{{{\boldsymbol{t}}_{1j}^{{}} } \mathord{\left/ {\vphantom {{{\boldsymbol{t}}_{1j}^{{}} } {\varpi_{1}^{11} }}} \right. \kern-0pt} {\varpi_{1}^{11} }}} & \cdots & {{{{\boldsymbol{t}}_{{1n_{1} }}^{{}} } \mathord{\left/ {\vphantom {{{\boldsymbol{t}}_{{1n_{1} }}^{{}} } {\varpi_{1}^{11} }}} \right. \kern-0pt} {\varpi_{1}^{11} }}} \\ \vdots & {} & \vdots & {} & \vdots \\ {{{{\boldsymbol{t}}_{i1}^{{}} } \mathord{\left/ {\vphantom {{{\boldsymbol{t}}_{i1}^{{}} } {\varpi_{i}^{11} }}} \right. \kern-0pt} {\varpi_{i}^{11} }}} & \cdots & {{{{\boldsymbol{t}}_{ij}^{{}} } \mathord{\left/ {\vphantom {{{\boldsymbol{t}}_{ij}^{{}} } {\varpi_{i}^{11} }}} \right. \kern-0pt} {\varpi_{i}^{11} }}} & \cdots & {{{{\boldsymbol{t}}_{{in_{1} }}^{{}} } \mathord{\left/ {\vphantom {{{\boldsymbol{t}}_{{in_{1} }}^{{}} } {\varpi_{i}^{11} }}} \right. \kern-0pt} {\varpi_{i}^{11} }}} \\ \vdots & {} & \vdots & {} & \vdots \\ {{{{\boldsymbol{t}}_{{n_{1} 1}}^{{}} } \mathord{\left/ {\vphantom {{{\boldsymbol{t}}_{{n_{1} 1}}^{{}} } {\varpi_{{n_{1} }}^{11} }}} \right. \kern-0pt} {\varpi_{{n_{1} }}^{11} }}} & \cdots & {{{{\boldsymbol{t}}_{{n_{1} j}}^{{}} } \mathord{\left/ {\vphantom {{{\boldsymbol{t}}_{{n_{1} j}}^{{}} } {\varpi_{{n_{1} }}^{11} }}} \right. \kern-0pt} {\varpi_{{n_{1} }}^{11} }}} & \cdots & {{{{\boldsymbol{t}}_{{n_{1} n_{1} }}^{{}} } \mathord{\left/ {\vphantom {{{\boldsymbol{t}}_{{n_{1} n_{1} }}^{{}} } {\varpi_{{n_{1} }}^{11} }}} \right. \kern-0pt} {\varpi_{{n_{1} }}^{11} }}} \\ \end{array} } \right]} \\ \end{array} } \\ \end{array} \begin{array}{*{20}c} \to \\ {} \\ \to \\ {} \\ \to \\ \end{array} \begin{array}{*{20}c} {\sum\nolimits_{j = 1}^{{n_{1} }} {{{{\boldsymbol{t}}_{1j}^{{}} } \mathord{\left/ {\vphantom {{{\boldsymbol{t}}_{1j}^{{}} } {\varpi_{1}^{11} = 1}}} \right. \kern-0pt} {\varpi_{1}^{11} = 1}}} } \\ \vdots \\ {\sum\nolimits_{j = 1}^{{n_{1} }} {{{{\boldsymbol{t}}_{1j}^{{}} } \mathord{\left/ {\vphantom {{{\boldsymbol{t}}_{1j}^{{}} } {\varpi_{i}^{11} = 1}}} \right. \kern-0pt} {\varpi_{i}^{11} = 1}}} } \\ \vdots \\ {\sum\nolimits_{j = 1}^{{n_{1} }} {{{{\boldsymbol{t}}_{1j}^{{}} } \mathord{\left/ {\vphantom {{{\boldsymbol{t}}_{1j}^{{}} } {\varpi_{{n_{1} }}^{11} = 1}}} \right. \kern-0pt} {\varpi_{{n_{1} }}^{11} = 1}}} } \\ \end{array}$$

A18 $${\boldsymbol{T}}_{c}^{*} { = }\left[ {\begin{array}{*{20}c} {{\boldsymbol{T}}_{11}^{*} } & \vdots & {{\boldsymbol{T}}_{1j}^{*} } & \vdots & {{\boldsymbol{T}}_{1m}^{*} } \\ \cdots & \vdots & \cdots & \vdots & \cdots \\ {{\boldsymbol{T}}_{i1}^{*} } & \vdots & {{\boldsymbol{T}}_{ij}^{*} } & \vdots & {{\boldsymbol{T}}_{im}^{*} } \\ \cdots & \vdots & \cdots & \vdots & \cdots \\ {{\boldsymbol{T}}_{m1}^{*} } & \vdots & {{\boldsymbol{T}}_{mj}^{*} } & \vdots & {{\boldsymbol{T}}_{mm}^{*} } \\ \end{array} } \right] = \left[ {\begin{array}{*{20}c} {{\boldsymbol{t}}_{11}^{*} } & \cdots & {{\boldsymbol{t}}_{i1}^{*} } & \cdots & {{\boldsymbol{t}}_{n1}^{*} } \\ \vdots & {} & \vdots & {} & \vdots \\ {{\boldsymbol{t}}_{1j}^{*} } & \cdots & {{\boldsymbol{t}}_{ij}^{*} } & \cdots & {{\boldsymbol{t}}_{ni}^{*} } \\ \vdots & {} & \vdots & {} & \vdots \\ {{\boldsymbol{t}}_{1n}^{*} } & \cdots & {{\boldsymbol{t}}_{jn}^{*} } & \cdots & {{\boldsymbol{t}}_{nn}^{*} } \\ \end{array} } \right]\begin{array}{*{20}c} \to \\ {} \\ \to \\ {} \\ \to \\ \end{array} \begin{array}{*{20}c} {\sum\nolimits_{j = 1}^{{n_{{}} }} {{\boldsymbol{t}}_{1j}^{*} = m} } \\ \vdots \\ {\sum\nolimits_{j = 1}^{{n_{{}} }} {{\boldsymbol{t}}_{ij}^{*} = m} } \\ \vdots \\ {\sum\nolimits_{j = 1}^{{n_{{}} }} {{\boldsymbol{t}}_{nj}^{*} = m} } \\ \end{array}$$

A19 $$M_{c}^{un} = (T_{c}^{*} )^{T} = \left[ {\begin{array}{*{20}c} {t_{11}^{*} } & \cdots & {t_{i1}^{*} } & \cdots & {t_{n1}^{*} } \\ \vdots & {} & \vdots & {} & \vdots \\ {t_{1j}^{*} } & \cdots & {t_{ij}^{*} } & \cdots & {t_{ni}^{*} } \\ \vdots & {} & \vdots & {} & \vdots \\ {t_{1n}^{*} } & \cdots & {t_{jn}^{*} } & \cdots & {t_{nn}^{*} } \\ \end{array} } \right] = \left[ {\begin{array}{*{20}c} {M_{11}^{un} } & \cdots & {M_{1j}^{un} } & \cdots & {M_{1n}^{un} } \\ \vdots & {} & \vdots & {} & \vdots \\ {M_{i1}^{un} } & \cdots & {M_{ij}^{un} } & \cdots & {M_{in}^{un} } \\ \vdots & {} & \vdots & {} & \vdots \\ {M_{n1}^{un} } & \cdots & {M_{nj}^{un} } & \cdots & {M_{nn}^{un} } \\ \end{array} } \right]$$

This structure serves as the basis for subsequent limit calculations and final weight determination.

*Step* 7*. Weighted Supermatrix for the Sub-criteria.*

The weighted matrix of the dimension is multiplied by the unweighted supermatrix of the sub-criteria to calculate the weighted supermatrix for the criteria, as shown in equation (A20).

A20 $$M^{w} = T_{\dim }^{w} \cdot M_{c}^{un} = \left[ {\begin{array}{*{20}c} {t_{11}^{{_{\dim }^{*} }} *T_{11}^{*} } & \vdots & {t_{i1}^{{_{\dim }^{*} }} *T_{i1}^{*} } & \vdots & {t_{m1}^{{_{\dim }^{*} }} *T_{m1}^{*} } \\ \cdots & \vdots & \cdots & \vdots & \cdots \\ {t_{1j}^{{_{\dim }^{*} }} *T_{1j}^{*} } & \vdots & {t_{ij}^{{_{\dim }^{*} }} *T_{ij}^{*} } & \vdots & {t_{mj}^{{_{\dim }^{*} }} *T_{mi}^{*} } \\ \cdots & \vdots & \cdots & \vdots & \cdots \\ {t_{1m}^{{_{\dim }^{*} }} *T_{1m}^{*} } & \vdots & {t_{im}^{{_{\dim }^{*} }} *T_{im}^{*} } & \vdots & {t_{mm}^{{_{\dim }^{*} }} *T_{mm}^{*} } \\ \end{array} } \right].$$

*Step* 8*. Computation Limiting Supermatrix.*

The weight supermatrix is raised to increasing powers until it converges, as shown in equation (A21).

A21 $$\user2{\mathcal{L}} = \mathop {\lim }\limits_{k \to \infty } \left( {M^{w} } \right)^{k} .$$

The resulting stable weight vector ${({M }^{w})}^{\infty }$ reflects the final importance of each semantic topic, incorporating both direct influence and network-level interactions. These weights provide a solid quantitative foundation for semantic–behavioral modeling and strategic analysis.
